# Process Performance and Operational Challenges in Continuous Crystallization: A Study of the Polymorphs of L-Glutamic Acid

**DOI:** 10.1021/acs.cgd.2c01424

**Published:** 2023-03-14

**Authors:** Ramona Achermann, Andraž Košir, Brigitta Bodák, Luca Bosetti, Marco Mazzotti

**Affiliations:** Institute of Energy and Process Engineering, ETH Zurich, 8092 Zurich, Switzerland

## Abstract

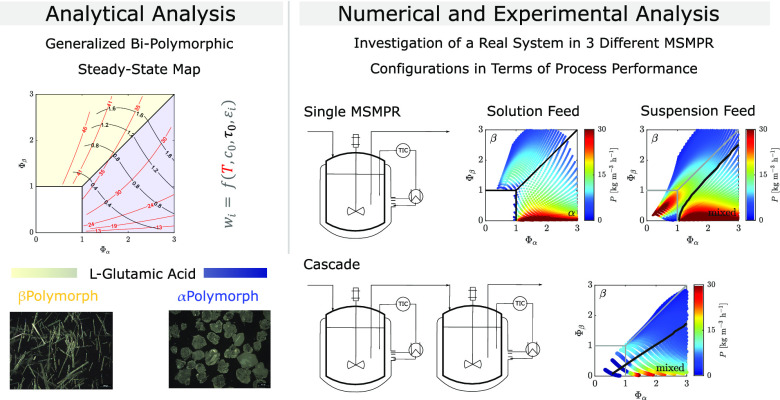

The crystallization of the two polymorphs of l-glutamic acid (LGA) is carried out in a continuous crystallization process, and its performance according to different criteria is evaluated. The study aims at identifying suitable operating conditions for producing either αLGA or βLGA with a high polymorphic purity. To this end, we investigate the process both from a theoretical perspective and through experiments using either a single stirred-tank crystallizer or a cascade of two stirred-tank crystallizers in series. In terms of theory, we extend the MSMPR-based steady-state stability analysis of Farmer et al. (Farmer, T. C. et al. AIChE J.2016, 62, 3505–3514) by accounting for the possibility of a nonrepresentative withdrawal of the solid phase from the crystallizer. Additionally, the process is simulated using population balance equations, thereby investigating the effect of operating conditions on polymorphic purity, yield, and productivity. Guided by the model-based conclusions, we identified suitable operating conditions and experimentally tested them. The experimental campaign has demonstrated that βLGA could be successfully and continuously produced in both process configurations according to the theory with performance as expected, whereas that was not possible for αLGA. The difference between the two stems from different operational challenges, whose consequence is that steady-state operation is attained in the case of βLGA but not in that of αLGA. In the former case, the needle-like βLGA crystals, which exhibit no agglomeration, tend to be only slightly oversampled; in the latter case, the prismatic αLGA crystals undergo major agglomeration and hence are very difficult to suspend and effectively withdraw from the crystallizer.

## Abstract 2

This work investigates the crystallization of the two polymorphs of L-glutamic acid in (non)ideal continuous stirred-tank crystallizer(s). A combination of experiments and modeling is used to evaluate the effect of the operating conditions on the process performance in terms of purity, yield, and productivity.

## 1 Introduction

Crystallization is an essential purification step in the production of active pharmaceutical ingredients (APIs).^2^ The majority of APIs can crystallize in various polymorphic forms. A polymorph can exhibit differences in crystal structure and consequently can also have different solubility, crystal shape, density, and other properties, thus affecting processability and bioavailability.^3^

Control of polymorphism is therefore crucial for designing a robust crystallization process, which produces a pure crystalline product of one polymorphic type while also achieving high yield and productivity. There are different strategies to produce the desired polymorph in a batch process^4,5^ as well as in a continuous process.^6−10^ When a continuous crystallization process is performed, e.g., in a mixed-suspension mixed-product removal (MSMPR), one can selectively produce either metastable or stable polymorphs, since the crystallizer is operated at a single solute concentration, thus avoiding solvent-mediated polymorphic transformation (SMPT). Moreover, continuous operation allows a higher flexibility and degree of process control and reduces costs.^11^ Which polymorph is formed depends on the interplay between thermodynamics and kinetics, i.e., the polymorphic-specific nucleation and growth rates. By manipulating the crystallization kinetics, e.g., by adapting the process operating conditions such as temperature, residence time, and feed concentration, one can produce a polymorph, which is otherwise not stable thermodynamically. This was shown in a landmark work by Farmer et al.,^1^ who reduced the complexity of dealing with bipolymorphic systems to the values of two dimensionless numbers. The approach has been supported by numerical tests and validated through experiments with different substances.^12−15^ This model has been extended further by adding agglomeration^16^ and breakage,^17^ thus showing that the polymorphic steady-state map can be adapted for more complex systems. Moreover, it has been shown that in a cascade of two MSMPRs, the seeds produced in the first crystallizer can be used to control the polymorphic outcome in the second crystallizer.^13,18^ By providing enough surface area of the desired form to the second crystallizer, it is possible to sustain its form at steady state.

To improve process control, the effect of a nonrepresentative withdrawal inside an MSMPR was previously studied, and an expression accounting for nonidealities was proposed.^19^ These nonidealities were modeled using computational fluid dynamics (CFD) tools. Decoupling the CFD and population balance equations (PBEs) model in such a way allows for much faster computation without loss of generality. This approach is especially valuable for the pharmaceutical industry and becomes important for systems such as the α-polymorph of LGA, the model compound studied in this work. LGA can crystallize in two polymorphic forms, (i) the metastable α l-glutamic acid (αLGA) and (ii) the monotropically stable β l-glutamic acid (βLGA), which has a lower solubility in the entire temperature range considered.^20^ αLGA crystals exhibit a prismatic shape, which is easy to filter and generally preferred for industrial purposes.^15,21,22^ However, crystals of that polymorph distribute heterogeneously within the crystallizer, and therefore, a representative withdrawal is operationally challenging.^19^ βLGA forms needle-like crystals, which are difficult to handle industrially and normally require further processing before usage, yet suspension heterogeneity is not an issue.^6^

The goal of this work is to produce both polymorphs of the model compound LGA with the highest attainable process performance, accounting for operational challenges for which both modeling and experiments are required. This work is divided as follows. section 2 provides the necessary tools needed for (i) the numerical part, which includes all the mathematical formulas of the population balance model, (ii) the dimensionless form of the PBE model used for the steady-state stability analysis, and (iii) the experimental part, describing the materials and methods. In section 3, the results obtained by the different models are discussed, namely, the results of the theoretical steady-state analysis performed with the dimensionless model and the results of the numerically resolved PBE model. The model-based considerations provide practical guidance for maximizing the process performance and serve as a bridge to the experimental campaign. The corresponding experimental results are described in section 4. Lastly, some concluding remarks are drawn in section 5.

## 2 Methods

Experiments and modeling are intertwined in this paper. The model equations based on the population balance equation framework are described in section 2.1, while section 2.2 defines the key performance indicators (KPIs) of a continuous crystallization process. Finally, the experimental methodology and materials are discussed in section 2.3, also providing some insight into the chosen analytical methods.

### 2.1 Model Equations

#### 2.1.1 General Population Balance Equations

For the sake of generality, we consider a cascade of well-mixed continuous crystallizers in series, modeled using population balance equations (PBEs), namely one for each polymorph *i* ∈ {α, β} in each of the stages *k* ∈ {1, ..., *n*_stages_}, with *n*_stages_ being the total number of stages.
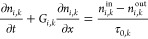
1In the equation above, *n*_*i*,*k*_^in^ is the number-based particle size distribution given in m^–4^, with *n*_*i*,*k*_^out^ and *n*_*i*,*k*_^in^ being the corresponding distributions at the outlet and at the inlet, respectively, of crystallizer *k*; *t* is time, *x* the characteristic crystal length, τ_0,*k*_ the nominal residence time in s (ratio of the suspension volume to the volumetric feed flow rate), and *G*_*i*,*k*_ the size-independent growth rate in m s^–1^. The boundary conditions (BCs) for the PBEs in eq 1 are defined as
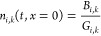
2with *B*_*i*,*k*_ corresponding to the nucleation rate in m^–3^s^–1^. The PBEs are coupled with the solute mass balance (MB) in each stage:
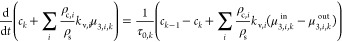
3In eq 3, *c*_*k*_ is the solute concentration in the liquid phase with the units g kg^–1^, ρ_c_, and ρ_s_ the crystal and solvent density, respectively, *k*_v,*i*_ the polymorphic crystal shape factor, and μ_3,*i*,*k*_ the third moment of the *i*-th polymorph distribution, with μ_3,*i*,*k*_^out^ and μ_3,*i*,*k*_^in^ being the corresponding moments at the outlet and at the inlet, respectively, of crystallizer *k*. The *j*th moment of distribution *n*_*i*,*k*_ is given by μ_*j*,*i*,*k*_ = ∫_0_^*∞*^*x*^*j*^*n*_*i*,*k*_ d*x*. Hence, μ_0,*i*,*k*_ is the number of crystals per unit solvent volume, and φ_*i*,*k*_ = *k*_v,*i*_μ_3,*i*,*k*_ is the volumetric fraction of the suspension occupied by the crystals, whereby ρ_c_*k*_v,*i*_μ_3,*i*,*k*_ represents the suspension density.

With neither additions nor withdrawals between crystallizers, the following equalities obviously hold: *n*_*i*,*k*_^in^ = *n*_*i*,*k*–1_^out^ for *k* ≥ 2. The quantity *c*_0_ is, in normal cases, a constant. Finally, note that the solute concentration in the liquid phase is mass based, whereas the particle concentration is volume based (hence, the term 1/ρ_*s*_ in the last equation).

To integrate the set of model equations in time, initial conditions (ICs) are needed, which can be written as follows:
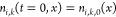
4
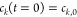
5Note that as seeds are present at *t* = 0, nucleation occurs exclusively via a secondary nucleation mechanism. Moreover, the ICs have no influence on the steady-state operation of any number of continuous crystallizers, provided that either a mixed polymorphic seed population is always chosen^15^ or the secondary nucleation rate expression for both polymorphs include the crystallization of the counter polymorph.

#### 2.1.2 Constitutive Equations

Rate expressions for growth, dissolution, and secondary nucleation for the two polymorphs of LGA were taken and adapted from the work of Hermanto et al.^23^ In these expressions, supersaturation of the *i*th polymorph (*i* ∈ {α, β}) is defined as
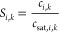
6

In the above equation, *c*_sat,*i*,*k*_ denotes the solubility of the *i*th polymorph under the operating conditions. The solubility expressions were taken from the work of Ono et al.^20^ The plotted solubility curves can be found in Figure 4.

The growth rate of αLGA can be written as
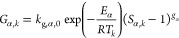
7whereas, the growth rate of βLGA is
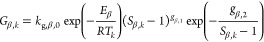
8Note that the unit of temperature in the growth rate expressions is kelvin. Furthermore, the dissolution rate of αLGA is defined as
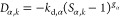
9The secondary nucleation rate of αLGA is defined as
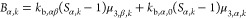
10and similarly, the secondary nucleation rate of βLGA is
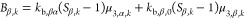
11

The values of all the kinetic parameters used in eqs 7–11 are given in Table 1. It is worth noting that a pure steady-state is not attainable anymore once a cross-nucleation term is introduced.^14^ However, the impact of the cross-nucleation term on purity is negligible for most process conditions considered in this study, and a high-purity steady-state can still be obtained. Finally, some additional considerations regarding the secondary nucleation rate expressions are provided in the Supporting Information (SI).

**Table 1:** Values of the Model Parameters for the Two Polymorphs of LGA.^15,23^

Mechanism	Parameter	Notation	Value
growth rate of α	pre-exponential factor	*k*_g,α,0_ (m s^–1^)	6.540
	activation energy	*E*_α_ (J mol^–1^)	4.3088 × 10^4^
	growth rate exponent	*g*_α_ (−)	1.859
dissolution rate of α	pre-exponential factor	*k*_d,α_ (m s^–1^)	3.5006 × 10^–5^
growth rate of β	pre-exponential factor	*k*_g,β,0_ (m s^–1^)	3.8387 × 10^22^
	activation energy	*E*_β_ (J mol^–1^)	1.7596 × 10^5^
	growth rate exponent	*g*_β,1_ (−)	1.047
	exponential constant	*g*_β,2_ (−)	0.778
nucleation rate of α	cross-nucleation factor	*k*_b,*αβ*_ (m^–3^ s^–1^)	1 × 10^2^
	homogeneous nucleation factor	*k*_b,α,0_ (m^–3^ s^–1^)	3.0493 × 10^7^
nucleation rate of β	cross-nucleation factor	*k*_b,*βα*_ (m^–3^ s^–1^)	7.2826 × 10^6^
	homogeneous nucleation factor	*k*_b,β,0_ (m^–3^ s^–1^)	4.8517 × 10^8^
material constants	shape factor α	*k*_v,α_ (−)	0.52
	shape factor β	*k*_v,β_ (−)	0.01
	density α	ρ_c,α_ (kg m^–3^)	1532
	density β	ρ_c,β_ (kg m^–3^)	1569

#### 2.1.3 Withdrawal Effects

When considering and modeling MSMPR crystallizers, “mixed-product removal” is typically assumed, i.e., the particle size distribution (PSD) at the outlet of the crystallizer is assumed to be identical to the one inside the crystallizer . However, depending on the position of the withdrawal tube and on the characteristics of the suspension, such representative removal might not be achieved.^19^ Although taking into account nonrepresentative withdrawal in a mathematical model adds complexity, it is necessary to enhance accuracy. To this purpose, the outlet PSD, *n*_*i*,*k*_^out^(*x*), can be defined as
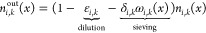
12where ε_*i*,*k*_ represents the polymorph-specific degree of dilution and δ_*i*,*k*_ the extent of the sieving effect, which is characterized by the size-dependent function ω_*i*,*k*_(*x*).^19^ On the one hand, since different polymorphs can crystallize in different shapes and grow at different rates, their dilution factors can differ as well; the higher its value, the lower the solid suspension density of the outlet stream and the longer the particles’ residence time. This may alter the nature of the steady-state attained. On the other hand, the sieving function describes additional nonidealities in the crystallizer related to a nonhomogeneous suspension density, which deviates from the ideal case, particularly in the case of larger crystals, that tend to settle. The former effect is easier to characterize, whereas the latter requires the definition of the function ω_*i*,*k*_(*x*); the former case will be considered first.

#### 2.1.4 Examples of Application

Equation 1 is the general formulation of a PBE for a cascade of continuous crystallizers, which can be specialized for application to several specific cases:

1. MSMPR crystallizer: *n*_stages_ = 1, *n*_*i*,1_^in^ = 0, *n*_*i*,1_^out^ = *n*_*i*,1_;
2. Continuous crystallizer with suspension feed and representative withdrawal: *n*_stages_ = 1, *n*_*i*,1_^in^ > 0, *n*_*i*,1_^out^ = *n*_*i*,1_;
3. Continuous crystallizer with either solution or suspension feed and nonrepresentative withdrawal: *n*_stages_ = 1, *n*_*i*,1_^in^ ≥ 0, *n*_*i*,1_^out^ ≠ *n*_*i*,1_;
4. Cascade of continuous crystallizers with or without representative withdrawal: *n*_stages_ > 1, *n*_*i*,1_^in^ = 0, *n*_*i*,*k*_^in^ = *n*_*i*,*k*–1_^out^ for *k* > 1, *n*_*i*,1_^out^ = *n*_*i*,1_ or *n*_*i*,1_^out^ ≠ *n*_*i*,1_ for *k* ≥ 1.

Some of these applications will be analyzed and discussed in the following.

#### 2.1.5 Numerical Solution

To solve the system of PDEs above, a high-resolution finite volume (HR-FVM) scheme has been implemented together with a van Leer flux limiter.^24,25^ The numerical solution is validated by comparison with the analytical solution, if possible. The analytical solution is only for cases featuring a single polymorphic crystal distribution at steady-state.^19^

### 2.2 Key Performance Indicators

Three KPIs are defined to assess the process performance in both experiments and simulations. They are the polymorphic purity, *w*_*i*_, the solute yield, *Y*, and the process productivity, *P*, i.e., a key product specification, and the measures of how well the material to be crystallized and the crystallization equipment are exploited.^17,26^ Yield and productivity can be considered proxies for operating costs and capital costs, respectively.

Purity is defined as the mass fraction of the target polymorph at the outlet of the cascade of crystallizers:
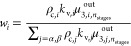
13

The yield *Y* is defined using the steady-state concentration of LGA in the liquid phase at the cascade outlet, , the concentration fed at the inlet of the first crystallizer *c*_0_, and the solubility of the stable polymorph at the conditions of the last stage :
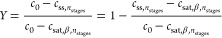
14The higher the yield, the lower , i.e., the more desupersaturated the system.^17^

Finally, the productivity *P* is defined as the mass of crystals harvested per unit volume and unit time at the cascade outlet (in this definition the masses of both polymorphs are considered):^15,17^
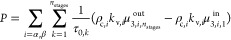
15It is worth noting that, by exploiting the steady-state form of the material balance of eq 3, one can recast the productivity in terms of the steady-state solute concentrations at the inlet and at the outlet of any cascade:
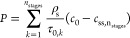
16

### 2.3 Materials and Methods

#### 2.3.1 Experimental Setup

**Figure 1:**
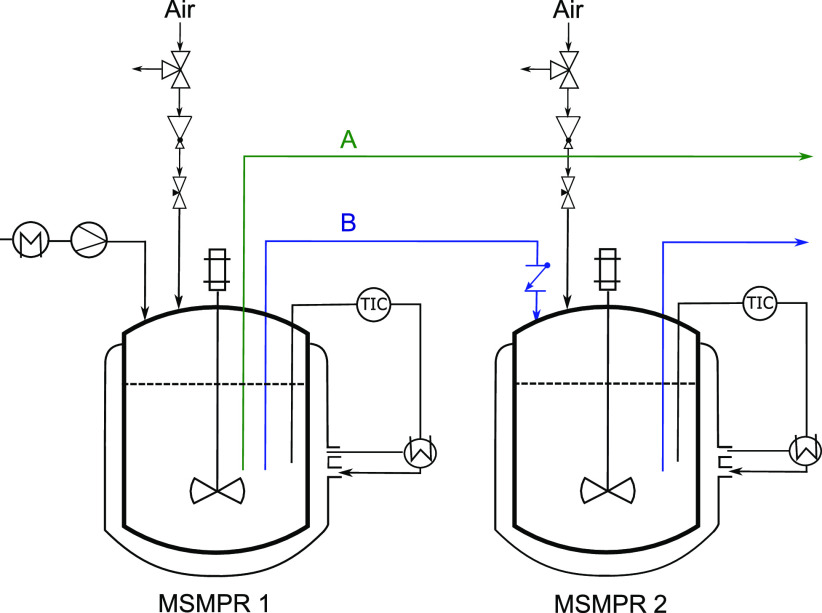
Flowsheet of the continuous crystallization setup used. For the operation including a single MSMPR, configuration A was employed (shown in green), and for the cascade operation, configuration B was used (shown in blue).

The setup used in this study is shown in Figure 1 and includes two MSMPRs in series, both with a working volume of 0.35 L (EasyMax 402, Mettler Toledo). The feed stream was fed using a peristaltic pump after being heated up to a high enough temperature (typically 60 °C in these experiments) to avoid primary nucleation and possible clogging. Depending on the type of experiment performed, either one MSMPR or two MSMPRs in series was used. The two operation protocols adopted in this study are indicated in Figure 1 with A and B, respectively. The suspension transfer from MSMPR1 to MSMPR2 and from MSMPR2 to the final collector tank was pressure-driven and occurred intermittently, as explained in detail elsewhere.^19^ A check valve was installed at the outlet of the MSMPR1 to avoid the backflow of air during the pressurization of the MSMPR2. A level control was installed in each crystallizer, which triggered the pressurization once a specified fill level height was reached.^26^ This avoided a suspension overflow in the crystallizer, whereas the pressurization time was kept short enough to keep the variation of the suspension volume below 10%.^27^ The whole setup was automated by using an Arduino microcontroller. Moreover, to ensure maximum control over the pressurization, i.e., the pressure applied and the flow rate induced), both a pressure reducer valve and a needle valve were installed.

#### 2.3.2 Experimental Protocol

An LGA solution of a given concentration *c*_0_ was prepared for each crystallizer as well as for the feed and heated to dissolve any crystals present in the solution. Then, the MSMPR vessels were cooled to the desired temperature, and the first MSMPR was seeded with the desired polymorph, thus avoiding unwanted primary nucleation, which also leads to encrustation.^7^ Note that a seed loading of less than 10% of the final mass produced was used. In the case of βLGA, the seed material was the one commercially available (Sigma-Aldrich, purity ≥99% HPLC), and in the case of αLGA, the seeds were produced via pH-shift,^21,28^ whereas particles <100 μm were chosen. After the seed addition, the process was started by activating the pump, which was previously set to the flow rate, corresponding to the desired nominal residence time. The outflow was initiated automatically once a certain fill level was reached. All experiments were repeated at least twice. For the experiments, which produce mostly αLGA, an anchor-shaped stirrer was used at a stirring rate of 250 rpm, resulting in less sedimentation of the particles, whereas for all other experiments a pitched blade was used at 500 rpm. At the end of the process, the shut-down procedure was initiated. Flushing of the feed line was carried out to avoid clogging, while the suspension was filtered, and the crystals were washed with ethanol, and then left to dry in the oven overnight.

#### 2.3.3 Analytical Methods

Due to the occurrence of fouling/encrustation, i.e., attachment of crystals on the measurement probes after a few hours, in situ measurements are difficult to achieve. Thus, the quantities needed to calculate the KPIs were obtained through offline measurements. The polymorphic purity was obtained via XRD measurements.^15,18^ To obtain quantitative information from the XRD, a calibration step was performed. Details on the construction of the calibration curve are provided in the Supporting Information. Moreover, optical microscopy images were taken for each sample. Finally, the evolution of the solute concentration over time inside the crystallizer, *c*_ss_, and the feed tank, *c*_0_, was measured gravimetrically. Similarly, the third moments of the distribution inside the crystallizer, , and in the outlet of the crystallizer,  were determined, i.e., by weighing the liquid and dried solid phase. Using these four quantities, one can determine whether the steady-state is reached by using the mass balance. At steady-state, a relative error between the solute depleted in the liquid phase (based on *c*_ss_) and accounted for in the solid phase (based on  and ) of less than 5% was deemed acceptable. Steady-state operation was normally obtained after around 7–10 residence times, whereas it is interesting to note that *c*_ss_ approaches a constant value before the steady state is reached.

## 3 Modeling Results

In this section, we carry out the model-based study of either one continuous crystallizer or two in series for a system where LGA is crystallized from an aqueous solution. To this aim, we solve the corresponding set of population balance equations presented in section 2.1. We report the results in section 3.2, with emphasis on the feasible operating conditions to obtain one polymorph or the other and on the KPIs attained, as defined in section 2.2. To frame it, this simulation-based analysis is preceded by an analytical derivation (see section 3.1), which extends literature results originally presented by Doherty and co-workers^1^ by accounting for nonrepresentative withdrawal.

### 3.1 Steady-State Stability Map

In this section, the analytical solution developed by Farmer et al.^1^ is extended by studying a single MSMPR (the stage index *k* is dropped for the sake of simplicity). The MSMPR is operated with a solution feed , under the assumption of a possibly diluted outlet stream, i.e.,  with ε_*i*_ ≥ 0 (δ_*i*_ = 0 in eq 12). With ε_*i*_ = 0 for both polymorphs, the case studied by Farmer et al.^1^ is recovered. Dilution increases the residence time, and a specific residence time for each polymorph, namely, τ_*i*_ = τ_0_/(1 – ε_*i*_), can be introduced.

In terms of constitutive equations, the following expressions for the growth and nucleation rates are used, in this subsection only:
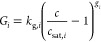
17
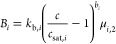
18Here, μ_*i*,2_ is the second moment of the *i*th PSD, and *c*_sat,*i*_ is the concentration of polymorph *i* at solubility. The constitutive equations used in this section differ from those presented in section 2.1, but they are consistent with those used by Farmer et al.^1^ and allow exploitation of a significant simplification of the resulting moment equations that stems from the use of the second moment of the PSD in the expression of the secondary nucleation rate in eq 18.^1^ The resulting system consists of seven ordinary differential equations (ODEs), instead of nine in the case where the third-order moments were used, as seen below in eqs 20–26, without losing anything in terms of generality. As a consequence, the solute balance of eq 19 is obtained by specializing the general solute balance of eq 3 to the case considered by Farmer et al.,^1^ which is made possible by the second moment dependence of the nucleation rate. The resulting solute balance, in the case of spherical particles considered here and by Farmer et al.^1^ where ρ_c,*i*_ = ρ_c_ for both polymorphs, reads:
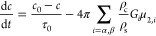
19Note that the liquid phase is not diluted at the outlet (i.e., *c*_out_ = *c*).

As detailed in the Appendix, section A.1, applying the method of moments to eq 1, by considering eqs 17 and 18,^29^ and normalizing transforms the set of partial differential equations (PDEs) into the following set of seven ODEs (with higher order moments needed only for a more accurate reconstruction of the PSDs)
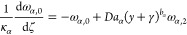
20
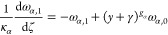
21
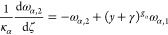
22
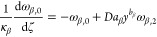
23
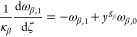
24
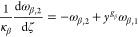
25
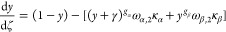
26where ω_*i*,*j*_ is the *j*th dimensionless moment of the PSD of polymorph *i*, *y* the normalized solute concentration (eq 41), and γ the normalized solubility difference between the two polymorphs (eq 41).

The Damköhler number *Da*_*i*_, defined as
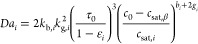
27includes the characteristic residence time as well as the characteristic crystallization time, which consists of nucleation and growth; it differs from the definition utilized by Farmer et al.^1^ only in the use of the polymorph-specific residence time. Note that the moment equations above also differ from those reported^1^ because of the presence of the dimensionless parameter κ_*i*_, i.e., one for each polymorph, which is the ratio between the nominal residence time τ_0_ and the residence time of the *i*th polymorph τ_*i*_, i.e., the term (1 – ε_*i*_).

For the general case, where ε_*i*_ ≠ 0 (τ_0_ ≠ τ_*i*_), the steady-state analysis is carried out in the same way as in Farmer et al.,^1^ i.e., by identifying the steady-states and by determining their stability through linearization around the steady-states and application of the Liénard–Chipart criterion to the coefficients of the characteristic polynomial of the associated Jacobian matrix and to the elements of the relevant Hurwitz matrix. The mathematical derivation is carried out in detail in the Appendix, section A.2. Note that, for the analysis, the α-polymorph is assumed to be metastable and the β-polymorph monotropically stable, as it is the case for LGA. The case ε = 0 and consequently τ_*i*_ = τ_0_ yields a special case of eq 27 that coincides with the definition in Farmer et al.^1^ and leads obviously to the same results.

**Figure 2:**
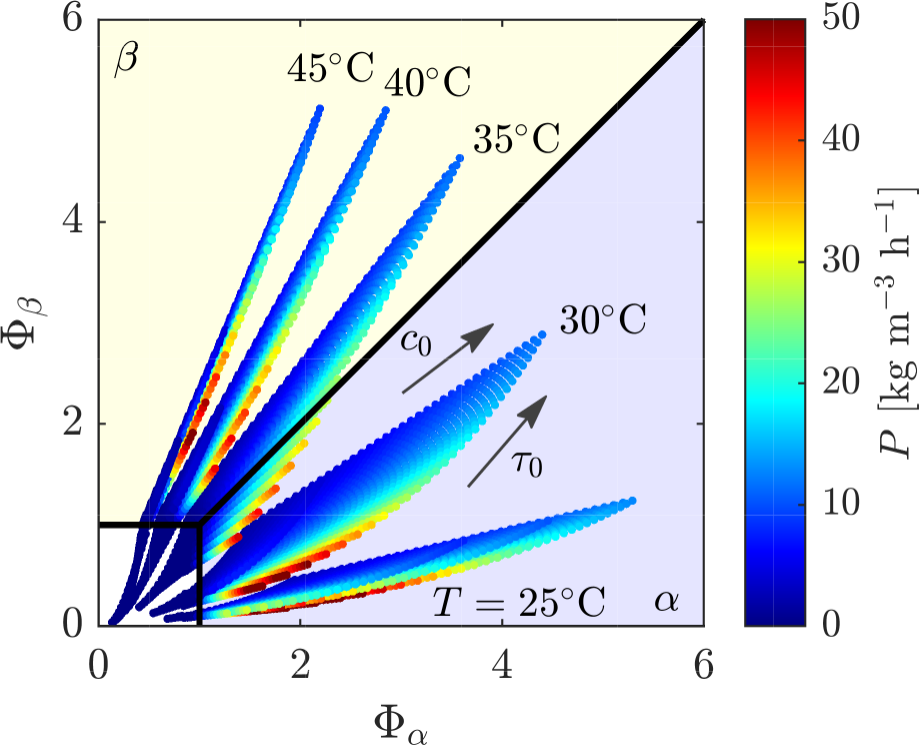
Effect of feed concentration, *c*_0_ = 20–40 g/kg, and residence time, τ_0_ = 0–2 h, on the productivity, *P*, plotted for five different operating temperatures, i.e., *T* = 25, 30, 35, 40, 45 °C.

Two remarks are worth making. First, the boundaries of the steady-state regions in the steady-state map shown in Figure 2 and in the following figures are the same as those in Farmer et al.^1^ This is true provided the extended definition of the Damköhler numbers given by eq 27 is used in the definition of the coordinates, Φ_α_ and Φ_β_ along the axes of such maps, namely:
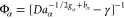
28
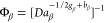
29

There are four steady-states for a bipolymorphic MSMPR system: (i) the trivial steady-state, whereby 0 < Φ_α_ < 1 and 0 < Φ_β_ < 1, where no crystals are present (given by the white square in the left bottom corner in Figure 2); (ii) the α steady-state (colored in a transparent blue), where 1 ≤ Φ_α_ and 0 < Φ_β_ < Φ_α_; (iii) the β steady-state (colored in a transparent yellow), where 1 ≤ Φ_β_ and 0 < Φ_α_ < Φ_β_; and (iv) the mixed steady-state, where both polymorphs are present, and 1 < Φ_α_ = Φ_β_; the mixed steady-state is the bifurcation between the two single polymorph steady-states. It is worth noting here that, as demonstrated in the Appendix, *y*_ss_ = Φ_α_^–1^ and *y*_ss_ = Φ_β_^–1^ in the α steady-state region and in the β steady-state region, respectively.

Thus, adding a dilution does not change the general features of the steady-states and their stability. Second, the advantage of this new analysis lies in the fact that accounting for different dilution of the two polymorphs (ε_α_ ≠ ε_β_) is automatically done by using correspondingly different values of the dilution factors ε_α_ and ε_β_ in eq 27. This is particularly relevant in the case where the two polymorphs have a different morphology, i.e., the case of LGA; the dilution factor of αLGA may well be larger than that of βLGA, i.e., ε_α_ > ε_β_.

### 3.2 Performance Analysis

In this section, the three process configurations indicated in the three columns of Table 3, namely, a single solution-fed continuous crystallizer (section 3.2.2), a single suspension-fed continuous crystallizer (section 3.2.3), and a cascade of two continuous crystallizers (section 3.2.4) are investigated.

#### 3.2.1 Operating Conditions and Operating Points

The corresponding operating conditions selected, in terms of values of *T*, τ_0_, *c*_0_, ε, are also reported in Table 3; note that in all simulations for the sake of simplicity the last parameter is kept constant at either ε = 0 (representative withdrawal) or ε = 0.5 (nonrepresentative withdrawal, with dilution of the withdrawn suspension).

Therefore, the three parameters *T*, τ_0_, and *c*_0_ define the operating conditions, but they are not enough to determine the operating point in the plane with dimensionless coordinates Φ_α_ and Φ_β_. The steady-state concentration *c*_ss_, which is an output of the simulation, is additionally needed, as explained below.

In order to position the numerical results in the dimensionless steady-state map, an algebraic transformation is needed, which is also employed in the literature.^16,17^ The kinetic expressions of Hermanto et al.^23^ were chosen. The growth rate expressions are
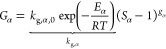
30
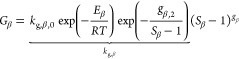
31In the case of the growth rates *G*_*i*_, the algebraic transformation step is straightforward. It is noteworthy that *k*_g,β_ in eq 31 depends on the supersaturation *S*_β_ and thus on the steady-state concentration *c*_ss_, which can only be calculated analytically for simple process configurations and is not known a priori. In the case of the nucleation rates *B*_*i*_, the third moment μ_3,*i*_ needs to be replaced by the second moment μ_2,*i*_, both being outputs of the simulation. This is done as follows:
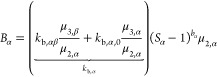
32
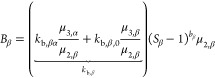
33In this way, the two nucleation rates obtain the same functional form as eq 17 and eq 18 with *b*_α_ = *b*_β_ = 1. Note that for each simulation the *k*_b,α_ and *k*_b,β_ would slightly change depending on the moments obtained. Moreover, even though the second and third moment of a polymorph at the steady-state of the counter polymorph is not zero (due to the presence of the cross-nucleation term), its value can be very small. Therefore, it was decided to use an average values for *k*_b,α_ and *k*_b,β_, reported in Table 2, which are based on the output of many simulation runs.^16,17^ The methodology applied seems to work well.

**Table 2:** Averaged Values Were Obtained for *k*_b,α_ and *k*_b,β_

Parameter	Values
*k*_b,α_ (m^–2^ s^–1^)	1.2e4
*k*_b,β_ (m^–2^ s^–1^)	6.5e4

For the suspension-fed crystallizer, both cases (i.e., feeding either a pure βLGA suspension or a pure αLGA suspension) are studied. For the cascade operation, however, the operating conditions in the first crystallizer are chosen so that a pure βLGA population is obtained and fed into the second crystallizer. It is worth noting that limitations on the maximum operating temperatures exist in a cascade; in general, *T*_2_ ≤ *T*_1_, with the equality being applied with the only purpose of increasing the residence time of the suspension in the system.

**Figure 3:**
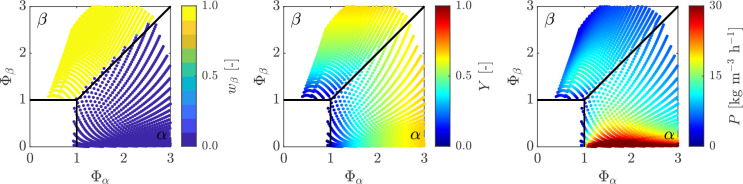
Heat maps showing purity of βLGA *w*_β_ (left), yield *Y* (middle), and productivity *P* (right) as a function of temperature *T* and residence time τ_0_ (see Table 3) for a single MSMPR operated with a representative withdrawal (ε = 0), while the inlet concentration is kept constant (*c*_0_ = 30 g/kg).

**Figure 4:**
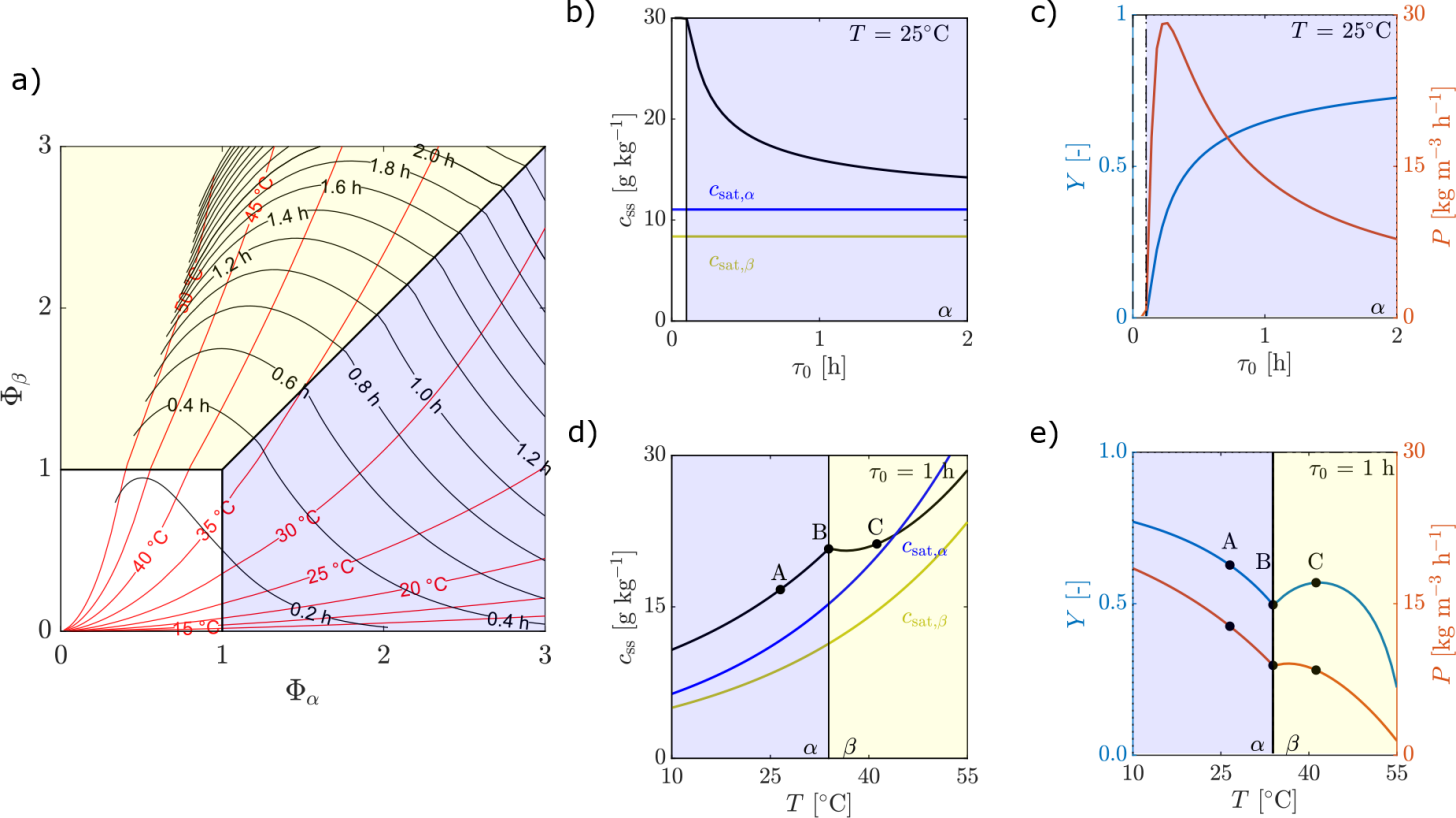
(a) Isotherms (in red; values are given in °C) and isolines of residence time (in black; values are given in h) on the dimensionless steady-state map. The effect of residence time on (b) steady-state concentration and (c) productivity, yield at constant temperature and the effect of temperature on (d) steady-state concentration and (e) productivity, yield at constant residence time are shown for a single solution-fed crystallizer. In panels (b) and (d), the solubilities of both polymorphs are provided additionally (*c*_sat,β_ in yellow and *c*_sat,α_ in blue). Note that the inlet concentration is kept constant (*c*_0_ = 30 g/kg).

**Figure 5:**
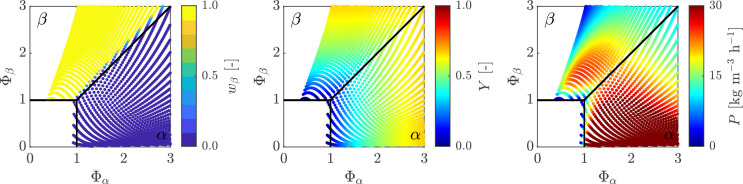
Heat maps showing the purity of βLGA *w*_β_ (left), yield *Y* (middle), and productivity *P* (right) as a function of temperature *T* and residence time τ_0_ for a single crystallizer operated with a diluted outlet stream (ε = 0.5, δ = 0). The inlet concentration is kept constant (*c*_0_ = 30 g/kg).

**Figure 6:**
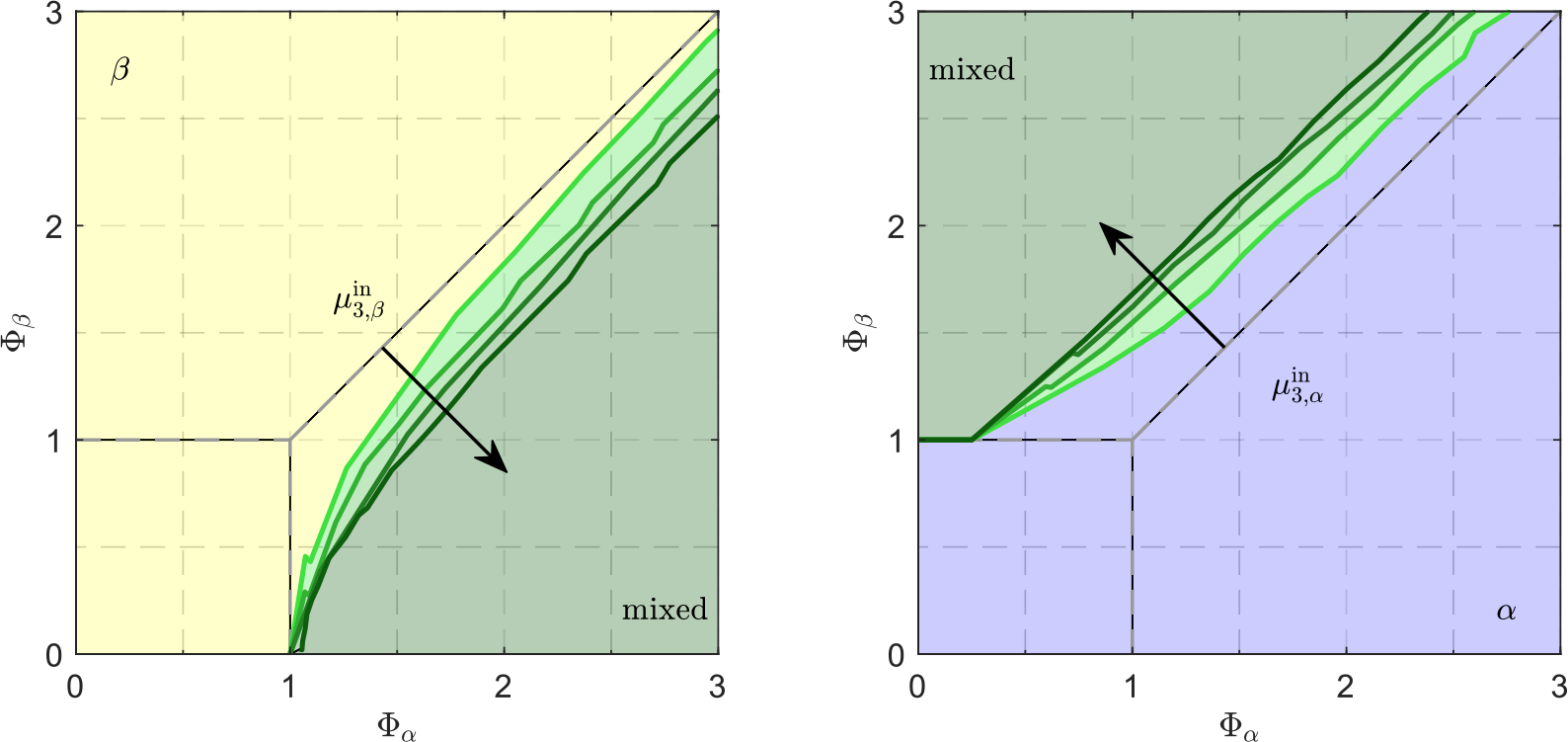
Change in the steady-state map boundaries in the case of continuously feeding a population of pure β crystals (left) and α crystals (right). The highest suspension density fed has an μ_3,α_^in^ = 0.018 for the α-polymorph and an μ_3,β_^in^ = 0.49 for the β-polymorph, respectively. The other μ_3,β_^in^ values are 25%, 50%, and 75% of the above reference value. Note that since a polymorphic pure suspension is fed to the MSMPR, only two steady-states are possible, i.e., the mixed steady-state (“mixed”, colored in a transparent green) and the pure steady-state of the polymorph being fed (β or α). The boundaries of the original steady-state map are shown with gray dashed lines for comparison. The green lines denote the 99% purity of the corresponding polymorph.

**Figure 7:**
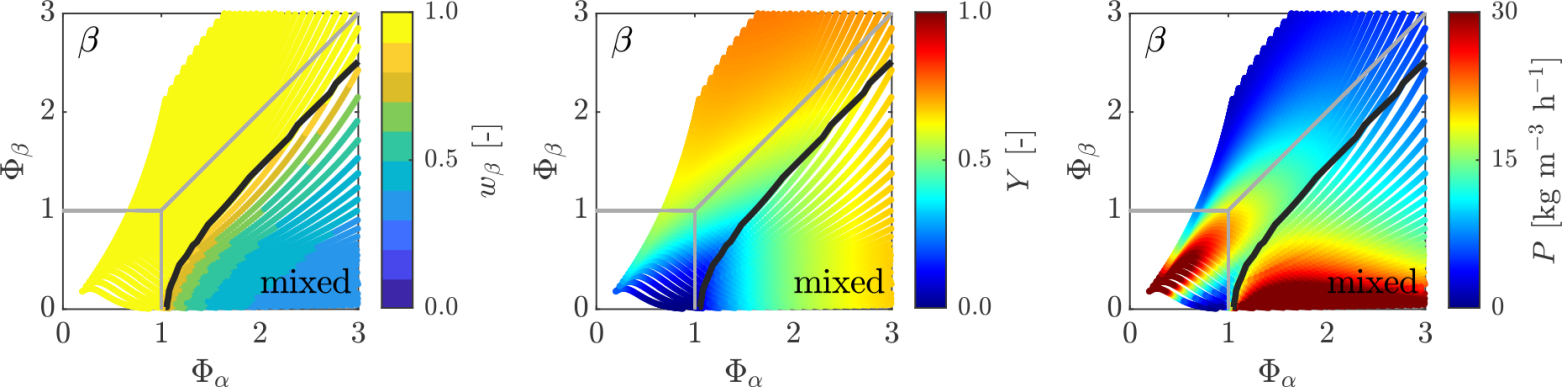
Heat maps showing purity of βLGA *w*_β_ (left), yield *Y* (middle), and productivity *P* (right) as a function of temperature *T* and residence time τ_0_ for a single MSMPR fed with a suspension of pure βLGA. The crystallizer is operated with a representative withdrawal (ε = 0, δ = 0). The steady-state map boundaries of a single MSMPR with a solution feed are shown in gray to ease comparison, whereas the black line denotes the 99% purity of βLGA.

**Figure 8:**
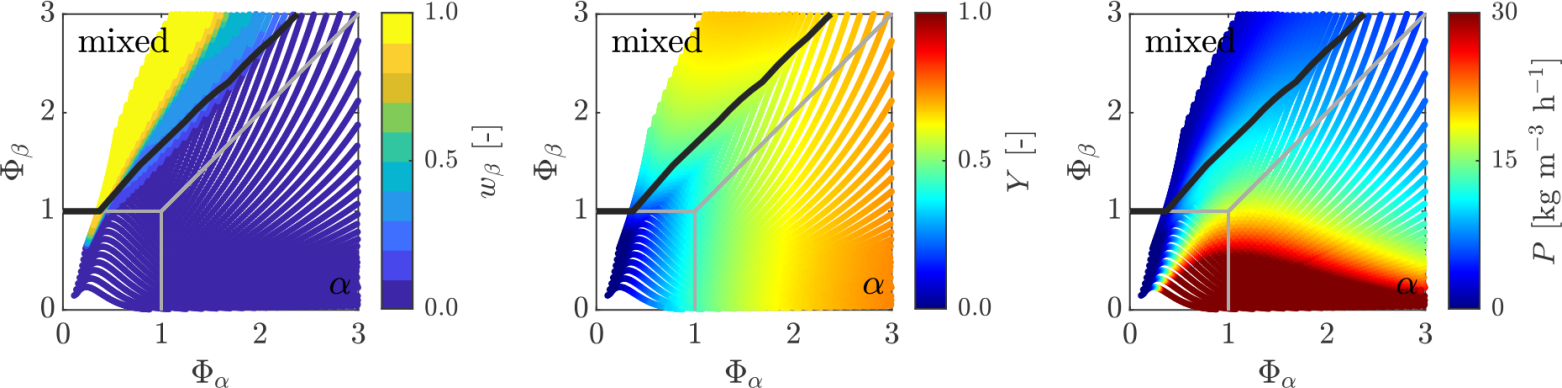
Heat maps showing purity of βLGA *w*_β_ (left), yield *Y* (middle), and productivity *P* (right) as a function of temperature *T* and residence time τ_0_ for a single MSMPR fed with a suspension of pure αLGA. The crystallizer is operated with a representative withdrawal (ε = 0, δ = 0). The steady-state map boundaries of a single MSMPR with a solution feed are shown in gray to ease comparison, whereas the black line denotes the 99% purity of αLGA.

For each operating point, the three KPIs, namely purity, yield, and productivity, are calculated. Results are presented as three distinct heat maps in the dimensionless (Φ_α_, Φ_β_) plane, i.e., one for each KPI; in all figures the boundaries of the steady-state regions calculated analytically in section 3.1 are also reported (pale gray lines), to help in the analysis of the results. The relevant figures are Figures 2–4 for a single solution-fed crystallizer with representative withdrawal, Figure 5 for a single solution-fed crystallizer with nonrepresentative withdrawal, and Figures 6–8 for a single suspension-fed crystallizer, with the feed being either a βLGA-suspension or an αLGA-suspension.

#### 3.2.2 Single Solution-Fed Continuous Crystallizer

The effect of changing the operating conditions for a single solution-fed continuous crystallizer with representative withdrawal, i.e., truly an MSMPR, is illustrated in Figure 2. For a given system and its set of physicochemical properties (solubilities, densities, nucleation, and growth rates, which are all function of temperature), the coordinates of the operating point in the steady-state map are calculated after choosing the process operating conditions.

In Figure 2, the operating points corresponding to five sets of operating conditions at five different temperatures, *T*, are plotted, while for each temperature the feed concentration, *c*_0_, and the residence time, τ_0_, are varied, as the arrows indicate. Each point has a color that indicates the corresponding productivity as to eq 15, according to the color code reported in the figure itself. It is worth noting that the steady-state attained for each operating point coincides with that expected based on the basis of its position in the steady-state map. This is not a trivial result, considering that the model used in the analytical derivation of the boundaries of the steady-state map (section 3.1) and that used in these simulations are not identical. This is, however, a reassuring result as both models are claimed in the literature to provide an accurate description of the LGA system, and therefore, in this context, they should provide very similar results.

We observe that low temperature levels favor the formation of the metastable polymorph, and vice versa. In principle, varying τ_0_ and/or *c*_0_ may drive the operating point from one region, e.g., the αLGA region, to the other, e.g., the βLGA region. In practice, for reasonable ranges of values of these two parameters, this does not happen at all temperatures, in fact, only at *T* = 35 °C in this case.

It can also be seen in the figure that productivity increases with an increase in *c*_0_ at any given temperature. Higher inlet concentrations, *c*_0_, led to higher supersaturation levels. Higher supersaturation levels, in turn, lead to higher nucleation and growth rates, which increase productivity. Note that *c*_0_ has a minor effect on which polymorph is obtained. Reducing the residence time, τ_0_, has an obvious positive impact on productivity; it is worth noting, however, that also changing τ_0_ has a minor effect on the final polymorph obtained. Because in real life operation of a crystallization process changing the feed concentration may be impractical, the following analysis will be carried out for a constant value of *c*_0_, in fact for *c*_0_ = 30 g/kg (see Table 3); we encourage the interested reader to conceptualize the effect of changing *c*_0_ by referring to Figure 2.

**Table 3:** Model Input Parameters Chosen for the Operation of a Solution-Fed Single Crystallizer, a Suspension-Fed Single Crystallizer, and a Cascade

			Cascade with two crystallizers	
	Single crystallizer solution-fed	Single crystallizer suspension-fed	*k* = 1	*k* = 2
*T* (°C)	10–50	10–50	45	10–40
τ_0_ (h)	0–2	0–4	0–4	τ_0,1_ = τ_0,2_
*c*_0_ (g/kg)	20–40	30	30	*c*_0,2_ = *c*_0,1_^out^
*V* (mL)	350	350	350	350
ε (−)	0, 0.5	0	0, 0.5	ε_2_ = ε_1_

For the case of a single MSMPR with a solution feed at *c*_0_ = 30 g/kg, the performance heat maps are presented in Figure 3, showing purity, yield, and productivity for a range of values of *T* and τ_0_ (see Table 3).

The purity heat map in Figure 3 (left) shows that the steady-state attained for each operating point coincides with that expected based on the basis of its position in the steady-state map obtained through the analytical derivation in section 3.1. The yield heat map (Figure 3, center) confirms that *Y* increases with increasing τ_0_, and shows that yield isolines are vertical and horizontal in the α and in the β-polymorph steady-state, respectively. This is a consequence of the property that the definition of yield, i.e., eq 14, can be written as *Y* = (1 – *y*_ss_), and of the fact mentioned above and discussed in the Appendix that the dimensionless solution concentration at steady-state is the reciprocal of the horizontal coordinate in the α steady-state (see eq 47) and the reciprocal of the vertical coordinate in the β steady-state (see eq 51). Finally, the productivity heat map in Figure 3 (right) demonstrates that while the βLGA polymorph can not be obtained at high productivity or at high yield, the αLGA polymorph can be obtained at high productivity at low temperatures, but a high yield also can be attained only for a small range of operating points (bottom right corner of the steady-state map).

The grid of operating points, whose corresponding KPIs are shown in Figure 3, are obtained at constant *c*_0_ = 30 g/kg by varying the temperature and nominal residence time as shown by the isolines in Figure 4a, namely, red lines at constant *T* (increasing from the bottom right corner to the top left corner) and black lines at constant τ_0_ (increasing from the bottom left corner to the top right corner). The effect of changing the residence time (at constant temperature, namely, *T* = 25 °C) and the temperature (at constant residence time, i.e., τ_0_ = 1 h) on yield, *Y*, productivity, *P*, and steady-state concentration in solution, *c*_ss_ is illustrated in Figure 4b–e; note that in Figure 4b,d also the solubilities of the two polymorphs are plotted for reference.

As τ_0_ increases at *T* = 25 °C the system goes from the trivial steady-state (no crystals) to the α-polymorph steady-state, while the steady-state concentration decreases and accordingly the yield increases because more supersaturation is consumed. Productivity exhibits in turn a maximum, because both numerator (proportional to *c*_0_ – *c*_ss_) and denominator (proportional to τ_0_) in eq 16 increase with τ_0_, but the numerator increases more than linearly with τ_0_ initially and then less than linearly as it asymptotically approaches the solubility of the prevailing polymorph. Note that the behavior would be somewhat different if the bifurcation line were crossed as τ_0_ varies, as in the case of the isothermal line at *T* = 35 °C (see below).

As *T* increases at τ_0_ = 1 h, the system transitions from the α-polymorph to the β-polymorph steady-state. Consequently, the relevant solubility level (Figure 4d) exhibits a discontinuity at the temperature on the bifurcation line (point B in the figure), corresponding to the switch from the α-polymorph to the β-polymorph solubility. At constant residence time, this leads to the angular point and to the nonmonotonic behavior of the steady-state concentration observed in Figure 4d and as a consequence of the yield and of the productivity (see Figure 4e).

Thus, summarizing, the rather peculiar behavior in the productivity heat map of Figure 3 can now be conceptualized as a combined effect of the decreasing trend of productivity with increasing temperature (at constant τ_0_) and of the presence of a sharp maximum for varying residence time (at constant *T*). A correspondingly high productivity island in the α-polymorph steady-state region and a less marked productivity maximum in the β-polymorph steady-state region are consequences of these combined effects.

Such effects are present also in the case of a single solution-fed continuous crystallizer (*c*_0_ = 30 g/kg) with nonrepresentative withdrawal (in fact in all cases that follow), whose performance heat maps are presented in Figure 5, for a range of values of *T* and τ_0_ (see Table 3; note that a value of ε = 0.5 has been used for all operating points).

The messages given by the purity and the yield heat maps in Figure 5 are the same as those for the case where ε = 0 in Figure 3. The productivity heat map in Figure 5 (right), however, shows that the longer effective residence times due to dilution of the outlet stream enable mid to high productivity levels also in the βLGA polymorph region (though the yield is still low). It is worth underlining (i) that this effect is remarkable, (ii) that it is amplified in the case where there is also a sieving effect, i.e., where ε = 0.5 and δ = 0.5(1 – ε) (the interested reader will find these results in the Supporting Information), and (iii) that our analysis, which includes both dilution and sieving effects in the withdrawal of the suspension, allows prediction of such important effects as well as interpretation of experimental results that could not be explained without considering nonrepresentative withdrawal. Lastly, it is interesting to note that comparing the productivity of different productivity heat maps at a given residence time and temperature provides additional information on the suspension density encountered at the crystallizer outlet, as they are directly proportional. Thus, in the case of dilution or sieving, higher suspension densities are encountered.

#### 3.2.3 Single Suspension-Fed Continuous Crystallizer

In this section, we analyze how the picture given in the previous section changes when the feed to the continuous crystallizer consists of a suspension instead of a solution, i.e., where *n*_*i*_^in^ ≠ 0. The strategy here is that of feeding the target polymorph in solid form (feeding both would make attaining a single polymorphic product impossible). This analysis is of interest both in the case of a single crystallizer if the suspension feed leads to better performances than the solution feed and in the case of a cascade where the suspension produced by the first crystallizer is fed to the second.

There are two aspects to this study. First, we will reconsider the steady-state map, as the one obtained for solution-fed continuous crystallizers cannot apply, at least for one reason: only the polymorph that is continuously fed in solid form can be obtained pure in the outlet suspension, and the other not. Second, we will calculate the process performances in the two cases of the feed in solid form of either one or the other polymorph using the detailed model and analyze them.

The PSD of the crystals fed into the continuous crystallizer is an additional operating choice. For the sake of simplicity, but without loss of generality, in this study, we assume the exponential PSD typically obtained in a solution-fed crystallizer, i.e., an MSMPR, at steady-state (which is the case of interest when considering a cascade):
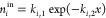
34The reference values of *k*_*i*,*j*_ are obtained by using the MSMPR functional dependence on *B*, *G*, and τ_0_, with the feed concentration being *c*_0_ = 30 g/kg, and the nominal residence time τ_0_ = 1 h, whereas *T* = 45 °C for a suspension feed consisting of a pure population of β-polymorph crystals and *T* = 25 °C for the pure α-polymorph case. In the following, we keep the values of *k*_*i*,2_ constant, while we vary those of *k*_*i*,1_, namely, by taking 25%, 50%, and 75% of the reference value, so as to explore the effect of different inlet suspension densities on the steady-state behavior of the suspension-fed continuous crystallizer.

In the case of a suspension-fed continuous crystallizer, the analytical steady-state analysis proposed originally by Farmer et al.^1^ and expanded in section 3.1 of this work cannot, to the best of our capabilities, be carried out, because of the presence of the inlet term in the PBEs. Because of its usefulness, we have calculated the steady-state map also for the suspension-fed continuous crystallizer using the detailed model; we have represented the results in the same (Φ_α_, Φ_β_) plane, and we have illustrated them in Figure 6, where also the boundaries indicated in the previous figures are again reported for reference.

The two diagrams in Figure 6 demonstrate that feeding a polymorphic pure suspension causes: (i) the disappearance of the steady-state region where the other polymorph is obtained pure as a solid product; (ii) the obvious disappearance of the region where no solids are formed, i.e., the square region with 0 < Φ_α_ < 1 and 0 < Φ_β_ < 1; (iii) the expansion beyond the gray straight line borders of the steady-state region, where the polymorph fed is obtained as a solid at high purity (such new boundaries in Figure 6 separate the high purity polymorph steady-state region—either yellow or blue—from the mixed state region—or green—where the final solid is a mixture of the two polymorphic forms; high purity here means that the purity of the target polymorph is at least 99%); and (iv) a clear effect whereby the larger the inlet suspension density, the larger the steady-state region, where the polymorph fed is obtained as pure solid product (note that the analytical case represented by the gray lines corresponds to the limit case where a suspension with zero solid density is fed to the crystallizer). The underlying mechanism leading to a change in the steady-state map lies in the fact that a suspension feed increases the surface and the volume of the crystals of the target polymorph, thus enhancing the secondary nucleation and the growth of its crystals. In other words, a suspension feed leads to a more pronounced depletion of supersaturation, hence *c*_ss_ is lower compared to the solution-fed crystallizer. A comparison between the solution-fed and the suspension-fed crystallizer therefore highlights identical qualitative trends of *c*_ss_, *Y*, and *P* in dependence on temperature and residence time. As Figure 4 shows, the difference in the quantitative trends, highlighting improved performance when feeding a suspension, is ultimately only on the density of the inlet suspension feed.

The main difference between the solution-fed and the suspension-fed crystallizer can be summarized as follows: with respect to the effect of temperature, namely, as explained in Figure 4, the steady-state map changes on the basis of the type of inlet suspension. In case the feed stream is a suspension of α, the temperature associated with the change of steady-state rises and the pure α region expands toward higher temperatures. Vice versa and simultaneously more applicable, when feeding a suspension of β, the stable β steady-state region expands toward lower temperatures.

The KPI heat maps for the cases of a βLGA polymorph and of an αLGA polymorph suspension-fed continuous crystallizer are presented in Figures 7 and 8, respectively, for a fixed inlet suspension density. In all six diagrams, the black solid line is the corresponding boundary separating the region where the polymorph fed is obtained at 99% purity from the mixed-state region. The purity heat maps in the diagrams on the left of both figures illustrate the results presented in Figure 6 from another perspective, i.e., by highlighting the purity level in the mixed region. The yield heat maps in the diagrams in the center of the two figures show that the isolines in the mixed steady-state regions and in the polymorphic pure steady-states are tilted. Further explanation and illustration of this effect can be found in the Appendix, section A.4).

The most interesting heat maps are those for productivity (diagrams on the right in Figures 7 and 8), which clearly show that the region of operating conditions where productivity is high expands away from the horizontal axis (compare Figures 7 and 8 with Figure 3). Moreover, in the case of the βLGA polymorph suspension-fed continuous crystallizer, a high productivity region appears, though at rather low yield, which is absent in the corresponding map in Figure 3. Note that higher productivities are a consequence of higher encountered suspension densities at the outlet, which operationally are more challenging to handle.

#### 3.2.4 A Cascade of Two Continuous Crystallizers

The cascade is a suspension-fed crystallizer realized in practice. The conclusions from the previous section regarding the suspension-fed crystallizer are thus fully applicable to the cascade configuration. Namely, apart from the expansion of the steady-state region in the second crystallizer of the polymorph being fed, a substantial improvement in yield accompanied by lower productivity levels is encountered as a consequence of an additional crystallizer, hence the higher total residence time (results are shown in the Supporting Information). Therefore, the suspension-fed single crystallizer clearly outperforms the cascade and the solution-fed single MSMPR in terms of productivity, whereas the cascade drastically favors operation in a higher yield.

## 4 Experimental Results

This section aims to fulfill multiple objectives. First, the experiments are designed to investigate the influence of different operating conditions and experimental configurations on the three KPIs, thus potentially validating the results of the theoretical analysis from section 3. Second, the experiments are used to quantify the degree of nonidealities in the suspension withdrawal step, i.e., one of the elements of novelty introduced in this work. Third, some of the operational challenges encountered along the way are discussed as well, while simultaneously providing possible solutions for some of these challenges.

**Figure 9:**
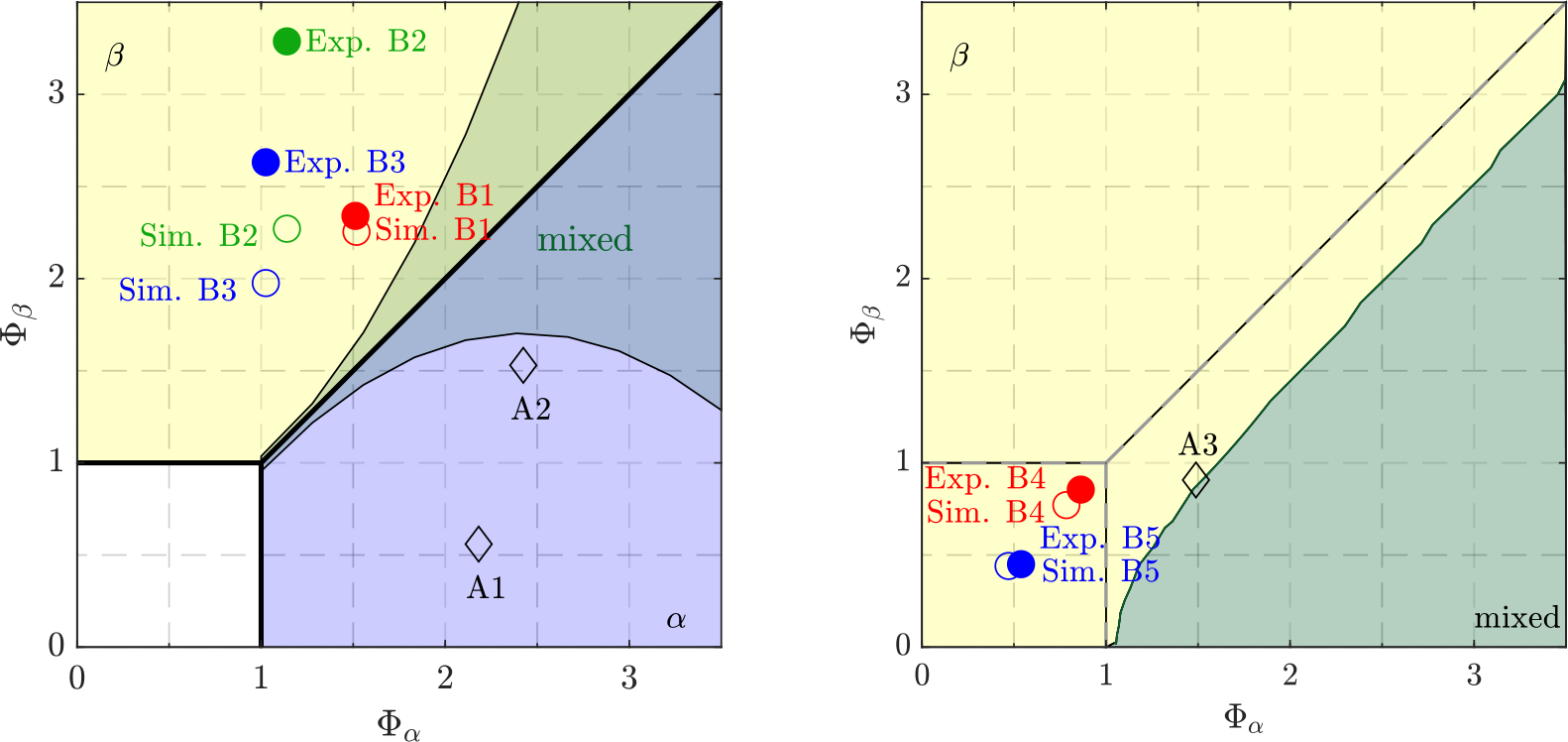
Experiments (filled markers) and simulations (empty markers) operated in a single MSMPR (left) and a cascade (right) were added to the dimensionless Damköhler plot. While the colored circles indicate experimental runs producing pure βLGA, the black empty diamonds indicate simulation runs where the production of αLGA was experimentally observed. The green shaded area, extracted from Farmer et al.^16^ indicates the boundary of a mixed steady-state region occurring when particles of LGA agglomerate. The boundary between the pure β-polymorph steady-state and the mixed steady-state is obtained when a suspension with μ_3,β,2_^in^ = 0.44 is fed.

The operational challenges are polymorph-specific since the two polymorphs of LGA differ significantly in their physicochemical properties and are therefore discussed separately. To this end, Experiments A1–A3, which result in the production of either pure αLGA or a mixed product, are evaluated first. However, these experiments exhibit difficulties in reaching steady-state. Then, Experiments B1–B5, leading to a stable and pure βLGA steady-state, are discussed next. In Figure 9, all of the experimental runs are positioned on the dimensionless steady-state map (left panel for the single solution-fed crystallizer operation and right panel for the cascade), with the colored filled and empty circles indicating the experiments and simulations for the βLGA trials, respectively, and the black empty diamond corresponding to runs, which lead to the production of αLGA (i.e., xperiment A3 was aimed at producing βLGA but resulted experimentally in a mixed steady-state). Note that for the latter, only the simulation runs are shown.

Some further considerations regarding Figure 9 should be made. The left panel of Figure 9, which includes the single crystallizer experiments, considers not only the nonrepresentative withdrawal but also the influence of agglomeration, extracted from the previous study conducted by Farmer et al.^16^ for LGA. Agglomeration was shown to lead to a modification of the steady-state map; i.e., the mixed steady-state close to the bifurcation expands as indicated by the green shaded region. The right panel of Figure 9 shows the experimental runs performed in a cascade; hence, it is constructed in the same way as Figure 6, namely looking only at the second crystallizer of a cascade, which is fed with the crystal population coming out of the first crystallizer (*c*_2_^in^ = *c*_1_^out^ and μ_3,β,2_^in^ = 0.44).

**Figure 10:**
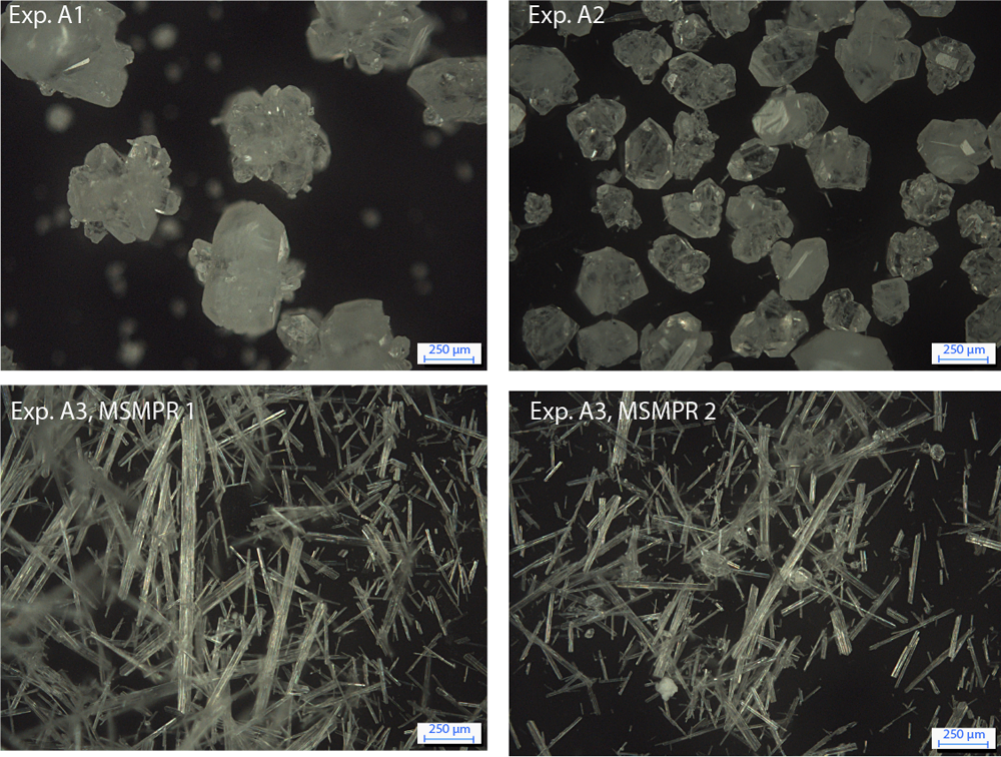
Top: Microscope images of the continuously produced αLGA in Exp. A1 (left) and Exp. A2 (right). Bottom: Microscope images of Exp. A3 for the first (left) and second (right) crystallizer. For each image, a scale corresponding to 250 μm is provided in the bottom right corner.

**Figure 11:**
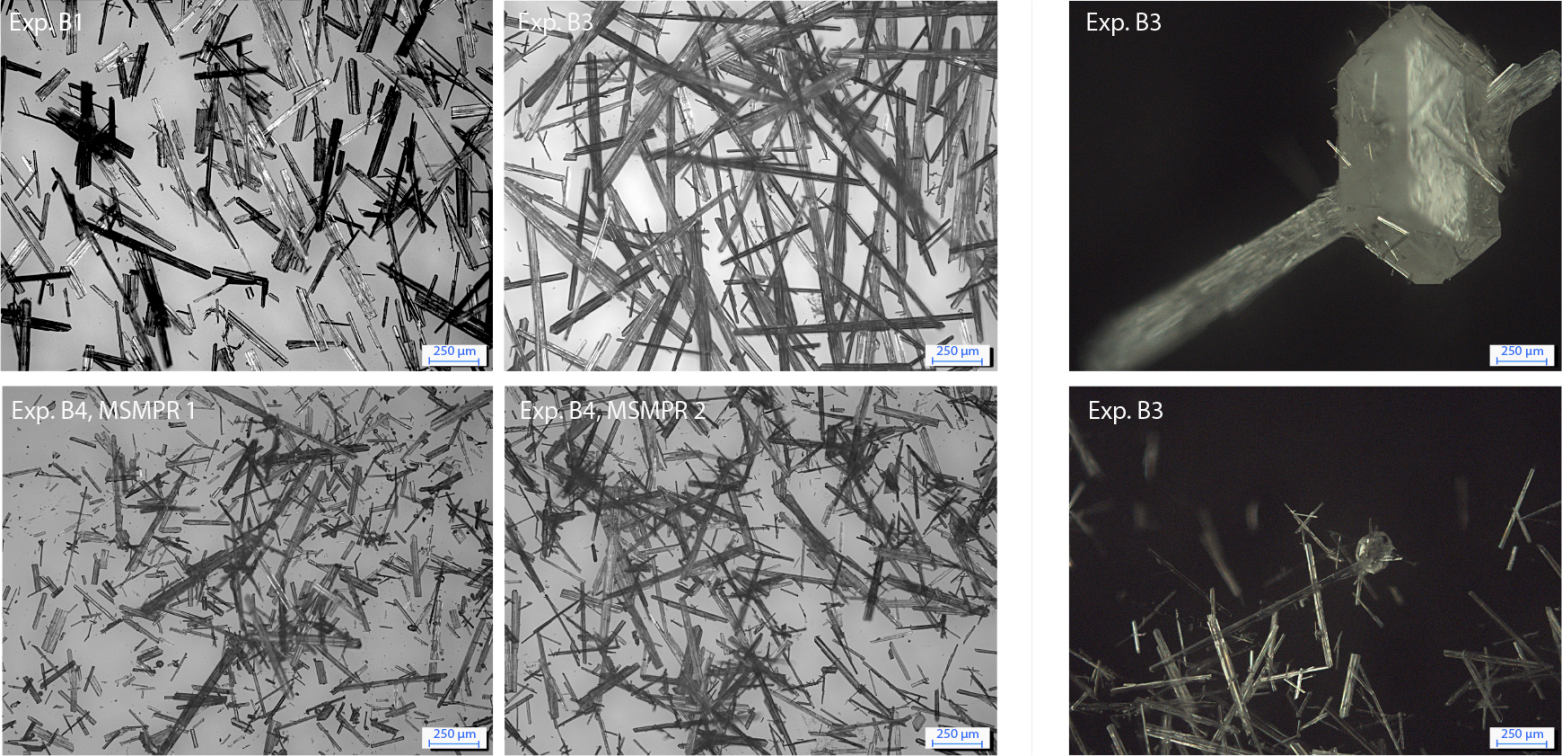
Top: Microscope images of the continuously produced βLGA in Exp. B1 (left) and Exp. B2 (right). Bottom: Microscope images of Exp. B4 for the first (left) and second (right) crystallizer. On the right-hand side, two microscope images showing a cross-nucleation event (Exp. B3) are captured, where a prismatic-shaped α-polymorph nucleates on the surface of a needle-shaped β-polymorph. For each image, a scale corresponding to 250 μm is provided in the bottom right corner.

A summary of all of the experimental results is provided in Tables 4 and 5, which list the operating conditions for experiments resulting in the production of the α- and β-polymorph, respectively. While a feed concentration of about *c*_0_ = 30 g kg^–1^ is used for all experiments, the temperature and residence time are varied. Furthermore, the tables also report the experimentally measured state variables, namely, the concentration in the liquid phase, *c*, for αLGA and at steady-state, *c*_ss_, for βLGA and the third moments inside the crystallizer, , and in the outlet of the crystallizer, . For all of the measured state variables, the average and its standard deviation are provided. Finally, the three KPIs, namely, purity given with reference to βLGA, denoted with *w*_β_, productivity, *P*, and yield, *Y*, are provided as well. Note that one can determine the supersaturation in the crystallizer by dividing the (steady-state) concentration by the solubility of βLGA provided in the caption of the corresponding table. Due to the distinct shapes of the two polymorphs, conclusions regarding the purity and the occurring crystallization phenomena of the obtained product can also be verified by examining the microscope images of the crystals. These microscope images are shown in Figure 10 for the experiments producing mostly αLGA and Figure 11 for the experiments producing βLGA.

**Table 4:** Summary of Operating Conditions and Experimental Results (Average Value ± Standard Deviation) For Three Experiments Used for Producing αLGA (Exps. A1 and A2) or Aimed at Producing βLGA, but Resulting in a Mixed Steady-State (Experiment A3)^a^

	Single solution-fed crystallizer		Cascade with two crystallizers
	Exp. A1	Exp. A2	Exp. A3
Operating conditions			
*T* (°C)	25	30	30
τ_0_ (min)	35	70	70
*c*_0_ (g/kg)	29.6 ± 1.9	29.7 ± 0.1	29.9 ± 0.7
State variables			
*c* (g/kg)	12.5 ± 0.1	14.2 ± 0.2	14.4 ± 0.5
μ_3,n_stages__ (−)	0.12 ± 0.007	0.09 ± 0.02	0.56 ± 0.01
μ_3,n_stagesout__ (−)	0.0069 ± 0.001	0.0056 ± 0.002	0.74 ± 0.03
KPI			
*w*_β_ (−)	0.04 ± 0.01	0.05 ± 0.02	0.82 ± 0.02
*Y* (−)	0.81 ± 0.01	0.79 ± 0.03	0.78 ± 0.02
*P* [kg/(m^3^h)]	0.54 ± 0.03	0.25 ± 0.05	7.5 ± 0.4

a Note that in the cascade experiment (Experiment A3), the temperature *T* and residence time τ_0_ are provided for the second crystallizer. The temperature in the first crystallizer was set to *T*_1_ = 45 °C, while the residence time was τ_1_ = τ_2_ = 70 min. The solubilities of βLGA at 25 °C and 30 °C are 8.4 g kg^–1^ and 9.9 g kg^–1^, respectively.

**Table 5:** Summary of Operating Conditions and Experimental Results (Average Value ± Standard Deviation) For Five Experiments Yielding a βLGA Steady-State (Experiments B1–B5)^a^

	Single crystallizer solution-fed			Cascade with two crystallizers	
	Exp B1	Exp B2	Exp B3	Exp B4	Exp B5
**Operating conditions**					
*T* (°C)	40	45	45	40	45
τ_0_ (min)	70	90	70	70	70
*c*_0_ (g/kg)	30.4 ± 0.4	29.8 ± 0.1	29.4 ± 0.1	29.2 ± 0.1	29.6 ± 0.2
**State variables**					
*c*_ss_ (g/kg) (exp)	20.8 ± 0.3	23.3 ± 0.1	23.7 ± 0.1	18.1 ± 0.3	20.4 ± 0.2
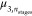 (−) (exp)	0.61 ± 0.01	0.39 ± 0.02	0.32 ± 0.05	0.66 ± 0.05	0.51 ± 0.02
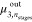 (−) (exp)	0.64 ± 0.03	0.42 ± 0.03	0.34 ± 0.02	0.70 ± 0.06	0.55 ± 0.02
*c*_ss_ (g/kg) (sim)	20.8	22.0	22.5	18.9	20.5
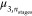 (−) (sim)	0.551	0.451	0.398	0.596	0.525
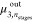 (−) (sim)	0.606	0.496	0.438	0.655	0.578
**Characterizing withdrawal**					
ε_β_ (−) (exp)	–0.05 ± 0.04	–0.04 ± 0.08	–0.06 ± 0.03	–0.09 ± 0.05	–0.07 ± 0.03
ε_β_ (−) (sim)	–0.1	–0.1	–0.1	–0.1	–0.1
**KPI**					
*w*_β_ (−) (exp)	0.97 ± 0.01	0.98 ± 0.01	0.97 ± 0.01	0.98 ± 0.01	0.97 ± 0.01
*Y* (−) (exp)	0.58 ± 0.02	0.48 ± 0.02	0.47 ± 0.01	0.73 ± 0.02	0.71 ± 0.01
*P* [kg/(m^3^h)] (exp)	8.59 ± 0.36	4.92 ± 0.89	4.78 ± 0.14	4.73 ± 0.42	3.68 ± 0.16
*w*_β_ (−) (sim)	1	1	1	1	1
*Y* (−) (sim)	0.58	0.59	0.54	0.68	0.70
*P* [kg/(m^3^h)] (sim)	8.15	5.19	5.89	4.41	3.88

a Experiments (denoted with “exp”) are directly compared to the simulation results (denoted with “sim”). For the simulations, a constant dilution factor value of ε = −0.1 was assumed. Note that in the cascade experiments (experiments B4 and B5) the temperature *T* and residence time τ_0_ are provided for the second crystallizer, whereas temperature and residence time in the first crystallizer are set to conditions in Exp. B3. The solubilities of βLGA at 40 °C and at 45 °C are 14.0 g kg^–1^ and 16.6 g kg^–1^, respectively.

### 4.1 Experimental Runs Producing αLGA

αLGA exhibits a few operational challenges preventing stable production at steady-state (at least in the employed experimental setup). Operational challenges include a high degree of agglomeration, a nonrepresentative withdrawal, and heavy encrustation (unwanted deposition of solid particles, especially due to a higher nucleation and growth rate at lower temperatures compared to the β-polymorph; further information is provided in the SI). The same conclusion is supported by the microscope images in Figure 10, where it is obvious that both experiments A1 and A2 produce agglomerates. The first two effects are coupled: as soon as particles reach a certain size, they settle, grow, and agglomerate. As a consequence, large and round crystals of millimeter size are obtained, which tend to remain in the crystallizer, as indicated by  in Table 4. Despite numerous attempts, including, e.g., changing the impeller blade, withdrawal remains highly nonrepresentative. Particles remain so long in the crystallizer that the value of the solute concentration in the crystallizer *c* approaches the solubility *c*_sat,α_, leading to a strong increase in the yield. This is, however, accompanied by a strong decrease in productivity, since particles cannot be withdrawn. Such a configuration resembles a batch operation and is not feasible in the context of continuous crystallization. Hence, further experiments producing αLGA were not considered.

For experiment A3, the model-based experimental design when assuming a representative withdrawal results in a pure β steady state, as shown in the right panel of Figure 9. However, after performing the experiment, a mixed steady-state was obtained, which can also be seen in the microscope images in Figure 10, wherein the second crystallizer crystals of the α polymorphic form are observable. Such a process is not useful in practice due to a lack of purity. Therefore, the focus is entirely switched to experiments producing pure βLGA.

### 4.2 Experimental Runs Producing βLGA

As opposed to experiments producing αLGA, multiple experiments producing pure βLGA result in a stable steady-state with reproducible outcomes in different repetitions (at least 2 repetitions for each type of experiment have been performed with very minor deviations between repeated experiments). High reproducibility is reflected in the low values of the standard deviation, most notably in *c*_ss_, μ_3_, and μ_3_^out^, which are all reported in Table 5. For instance, the highest standard deviation of *c*_ss_ is observed in experiment B1 and amounts to (only) 0.3 g/kg. A higher variability is observed for measurements of μ_3_ and μ_3_^out^, which are more prone to experimental errors.

As shown in the microscope images in Figure 11, βLGA crystallizes in a needle-like morphology and therefore poses a significant risk of process disruption in a continuous crystallizer. In such conditions, clogging is a prominent issue in the setup, which is amplified by adding additional elements to the pipelines, such as valves or pumps. In order to limit clogging, using a pressure-driven withdrawal with a large diameter tube is identified as the most effective measure. Nonetheless, clogging still occurs in some experimental tests; thus, manual flushing and declogging of the process lines remain necessary, especially in the cascade operation, where additionally higher suspension densities are encountered (see the values of  and  reported in Table 5). Even though the measured purity is very high for all of the experimental runs, it is interesting to note that cross-nucleation events are often observed. Cross-nucleation is a form of heterogeneous nucleation, where one polymorph nucleates in the presence of crystals of another polymorph, possibly on them (more information on this topic can be found in the Supporting Information).^2,30^ Direct evidence of such a cross-nucleation event is shown in the right-most microscope images of Figure 11, where a prismatic αLGA crystal nucleated on the surface of a needle-shaped βLGA crystal.

To continue the discussion with yield and productivity, one must take a closer look at the measured state variables in Table 5. From the two moments,  and , one can determine the polymorph-specific dilution factor ε_*i*_, which characterizes how representative the withdrawal is^19^
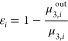
35From Table 5, one can see that a negative dilution factor is measured (i.e., corresponding to the concentration of the suspension in the withdrawal stream), meaning that βLGA crystals are slightly oversampled. In other words, the needlelike βLGA crystals remain in the crystallizer for less time than τ_0_, therefore lowering the yield compared to the ideal MSMPR operation, where withdrawal is representative, i.e., where ε_β_ = 0. However, due to the variations of the experimental measurements, a value of ε_β_ ≈ – 0.1 was determined and used in the simulations. Moreover, both the suspension density inside the crystallizer and in the outlet are also lower as compared with the ideal MSMPR, which leads to a reduction in productivity. Similar trends, which might point to the same operational challenges, and oversampling of βLGA crystals are also observable in the literature,^18^ though the analysis, rationalization, and characterization of the phenomena involved are novel in this work.

However, comparing the single crystallizer with the cascade operation, in which the second crystallizer is operated at lower temperatures, shows a clear improvement of yield by roughly 55% (experiment B3 vs experiment B4). The advantage of operating at lower temperatures and lower residence times is also apparent in the single crystallizer when comparing experiment B1 to experiment B2 and finally to experiment B3: The third moments and thus also the yield and productivity all decrease in the same order as in these three experiments. Nevertheless, despite the improvement of yield and productivity when decreasing the operating temperature and residence time in experiment B3, the operating point can approach the mixed steady-state region in the case of small process disturbances (see the position of experiment B3 in Figure 9). Hence, purity can be affected under these conditions, and therefore, operating the crystallizer further away from the mixed steady-state region is recommended.

## 5 Concluding Remarks

In this work, a performance evaluation of a continuous crystallization process aimed at selectively producing one of the LGA polymorphs in different operating configurations is conducted. The model-based analysis, which accounts for a nonrepresentative size-independent solid withdrawal in a continuous stirred tank crystallizer, provides a sound understanding of the relationship between polymorphic purity and a certain set of process parameters, i.e., temperature, residence time, inlet concentration, and polymorphic dilution factor. Moreover, including the polymorphic dilution factor in the analytical steady-state analysis, a novelty of this work, leads to a more general formulation of the polymorphic steady-state map, thus expanding its applicability. In this updated map, the Damköhler number, *Da*_*i*_ (*i* = α, β), becomes a function of the polymorphic residence time τ_*i*_, which varies in response to the polymorph-specific nonrepresentative withdrawal, associated with the heterogeneity of the suspension density within the crystallizer. A nonhomogeneous suspension density stems both from hydrodynamic considerations and from different flowabilities of individual polymorphs (related to their morphology).

Furthermore, the conclusions of the steady-state analysis are further supported with a PBE model, which was developed to assess process performance not only based on the basis of purity but also with respect to two other KPIs, namely, yield and productivity. The developed concepts and conclusions are general and applicable to any substance exhibiting polymorphism; however, in the scope of this work, they are applied to l-glutamic acid. Moreover, three process configurations, namely, a solution-fed crystallizer, a suspension-fed crystallizer, and a cascade, were evaluated. The simulation results demonstrate that the suspension-fed crystallizer maximizes productivity, whereas the cascade operation improves yield due to an additional crystallizer. Considering a nonrepresentative withdrawal though has a more complex effect on the process performance. Depending on the type of nonrepresentative withdrawal, the performance might be improved if the particles are undersampled and thus remain longer in the crystallizer (i.e., higher effective residence time). Conversely, in the case of oversampling, productivity and yield actually decrease since particles have less time to grow.

These conclusions were then investigated experimentally. First of all, polymorph-specific operational challenges were identified. In experiments aimed at producing αLGA heavy agglomeration and encrustation prevented achieving a stable steady-state operation, whereas stable steady-states, where pure βLGA crystals were produced with high reproducibility, were demonstrated. In the case of βLGA experiments, conclusions closely correspond to the model predictions; namely, increasing the residence time leads to a depleted supersaturation and increased suspension density. Handling needle-like crystals and coping with clogging were deemed the main operational challenges associated with βLGA, which was successfully mitigated with a pressure-driven withdrawal.

Finally, the developed methodology allows quantification of the nonrepresentative withdrawal. In the βLGA experimental trials, the dilution factor was found to be ε_β_ ≈ −0.1 for all experiments, indicating a slight oversampling of the needlelike βLGA crystals, which closely follow the flow field in the crystallizer. Hence, the developed approach represents a useful tool to capture and describe a more realistic continuous crystallization process, which can be employed for the purposes of process design, optimization, troubleshooting, and intensification.

## Figures and Tables

**Figure 12:**
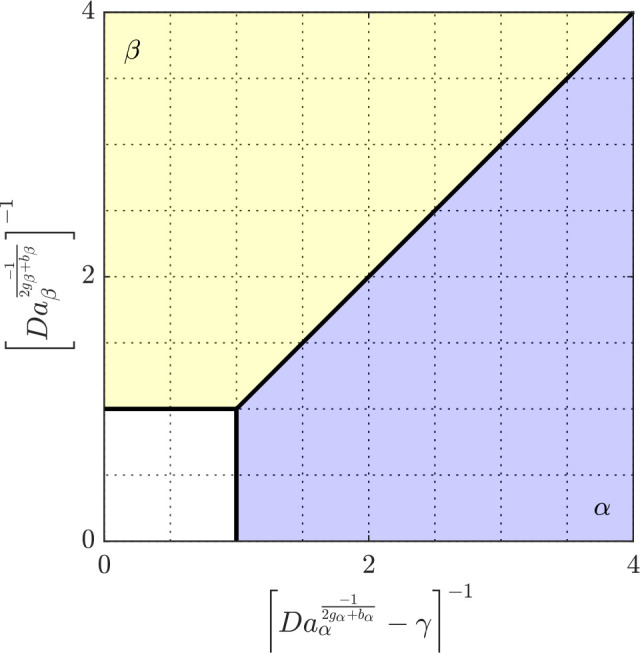
Polymorphic steady-state map operated under an outlet stream, which is diluted (0 ≤ ε_*i*_ ≤ 0.5, δ = 0) or concentrated (−0.5 ≤ ε_*i*_ ≤ 0, δ = 0) in solid particles.

**Figure 13:**
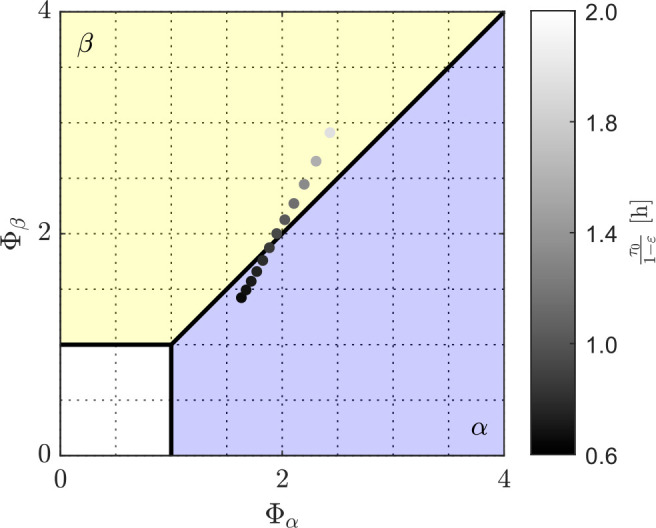
Bipolymorphic steady-state map. The operating points associated to different sets of operating conditions are plotted as gray-shaded round markers for varying values of τ_0_/(1 – ε) (see the color bar). Along the gray arc, *T* = 34 °C and *c*_0_ = 30 g/kg are constant.

## Appendix

### A.1 Method of Moments and Nondimensionalization

Introducing a specific residence time for each polymorph (τ_*i*_ = τ_0_/(1 – ε_*i*_)) leads to the following PBEs:

36

Applying the method of moments to eq 36,^29^ where the general form of the *j*th moment of the polymorph *i* is μ_*i*,*j*_ = ∫_0_^*∞*^*x*^*j*^*n*_*i*_(*x*,*t*) d*x*, transforms the set of PDEs into ODEs.

37

38

The first three moment equations are needed to fully characterize the steady-state of a system. As a next step, the above equations will be made dimensionless. For this the following variables are introduced

39

40

41

42

43

44

45

46

with . The resulting dimensionless sets of ODEs are given by eqs 20–26. These model equations differ from the original ones presented and analyzed by Farmer et al.^1^ in two aspects. Firstly, in this work, the withdrawal effects are considered by introducing κ_α_ and κ_β_, and secondly, the definition of the Damköhler numbers in eq 27 is slightly different, again due to the consideration of withdrawal effects.

### A.2 Steady-State Analysis

A steady-state is defined when the system does not change anymore with respect to time. The steady-state map, given by Figure 12, is obtained for each of the four steady-states as follows.

#### Trivial Steady-State

The trivial steady-state, where ω_α_ = ω_β_ = 0 and *y* = 1 corresponds to no nucleation and growth (either under-saturated solution or very fast residence times). Seed crystals, which were initially present, are completely washed out after several residence times.

#### α Steady-State

In the α steady-state (ω_α_ ≥ 0, ω_β_ = 0, *y* ≤ 1), eqs 20–26 can be simplified to the following four steady-state values:

47

48

49

50

#### β Steady-State

Analogously, in the β steady-state (ω_α_ = 0, ω_β_ ≥ 0, *y* ≤ 1), eqs 20–26 simplify as follows:

51

52

53

54

#### Mixed Steady-State

The mixed steady-state lies on a bifurcation at which the *y*_ss_ of both polymorphs are equal. This line is described by the following equation:

55

The general Jacobian matrix of the system is given below:

To analyze the stability of each steady-state, the matrix problem is first converted into a polynomial problem (eq 56). The characteristic polynomial of the matrix is shown in eq 56 with *n* = 7 and λ being the eigenvectors. Then, the Liénord–Chipart criterion is applied, which states that all even Hurwitz determinants  of that matrix as well as all even constants of the characteristic polynomial equation *a*_*n*_ have to be positive.^31,32^

56

For the trivial steady-state, the following seven constants of the characteristic polynomial equation *a*_*n*_ and three determinants are obtained:

57

58

59

60

61

62

63

64

*D*_4_ and *D*_6_ are unsuitable for print, and thus, omitted here. However, one can see that in case τ_α_ = τ_β_ = τ_0_, the above expressions (including the expressions obtained for *D*_4_ and *D*_6_) become identical to the one stated in Farmer et al.^1^ Thus, it comes at no surprise that the same steady-state conditions are obtained. In order to have positive values for *a*_*n*_ and *D*_*k*_ (*a*_*n*_ > 0, *D*_*k*_ > 0) and a stable trivial steady-state, the following two inequalities need to be fulfilled:

65

66

The region within the steady-state map for which eqs 65 and 66 are fulfilled is indicated in the bottom left part in Figure 12 by the white square.

For the two pure crystal steady-states, i.e., the α and β steady-state, the following four variables for the two polymorphs are introduced:

67

68

With this, the seven constants of the characteristic polynomial for both polymorphs as well as the second Hurwitz determinant *D*_2_ are presented. Note that, since the expressions for the different values of *a*_*k*_ are the same for both polymorphs, they are written with the general indices *i*, whereas an index *j* refers to the other polymorph.

69

70

71

72

73

74

75

76

77

The other two Hurwitz determinants *D*_4_ and *D*_6_ are not depicted as they do not qualitatively change the outcome.^1^ In case, these two tests are not fulfilled, the solution oscillates around the steady-state, which was predicted to be stable by the other tests.^1^ To obtain a stable steady-state, all the above tests have to be positive. *a*_1_–*a*_7_ become positive when both η_*i*_ > 0 and ϕ_*i*_ > 0. This is given when the following four conditions, provided by eqs 78 and 79, are fulfilled:

78

In eq 78, the inequality for α is shown on the right side of the trivial steady-state and the condition for β above the steady-state region, as can be seen in Figure 12.

79

The condition for the α-polymorph in eq 79 corresponds to the area below the α/β bifurcation (indicated with a transparent blue in Figure 12) and the condition for the β-polymorph to the area above the bifurcation (indicated with a transparent yellow in Figure 12). Knowing that *Da*_*i*_ > 0, ζ_*i*_ > 0 and 0 < ϕ_*i*_ < 1 means that *D*_2,*i*_ > 0.

Note that the boundaries of the steady-state regions in the steady-state map shown in Figure 12 are the same as in Farmer et al.;^1^ this is true provided the extended definition of the Damköhler numbers given by eq 27 is used.

### A.3 Influence of Dilution

The influence of dilution on the bipolymorphic steady-state map is shown in Figure 13.

Looking at the arc with gray-shaded symbols, these are obtained by keeping *T* and *c*_0_ constant (*T* = 34 °C and *c*_0_ = 30 g/kg) and changing either τ_0_ or ε (we are considering ε = ε_α_ = ε_β_ here; see the color bar for the values of the ratio τ_0_/(1 – ε)). Increasing either τ_0_ or ε increases *Da*_*i*_, hence causing the operating point in the steady-state map to move along the arc from the region where the α-polymorph is formed to where the β-polymorph is obtained. Increasing the residence time either by changing the nominal residence time or the withdrawal dilution increases the effective residence time of the crystals in the crystallizer, thus favoring the more stable polymorph. The whole steady-state analysis performed is also valid for ε_α_ ≠ ε_β_.

### A.4 Feeding a Suspension

When feeding a suspension of the β-polymorph, the expression for the dimensionless solute concentration *y*_ss_ at the β steady-state becomes a complex functions of the polymorphic’s corresponding moments:

80

81

82

83

The same problem occurs in the α steady-state when feeding the α-polymorph

84

85

86

87

with ω_*i*,*j*_^in^ representing the *j*^th^ dimensionless moment of the PSD of polymorph *i* at the inlet of the crystallizer.

## Supporting Information Available

Additional details regarding the population balance equation (PBE) model; simulation results and discussion of a single solution-fed crystallizer when sieving is considered; simulation results and discussion of a cascade operation; details regarding the XRD calibration; considerations regarding process risks associated with fouling (PDF)
