# Supplementary material for: Process Performance and Operational Challenges in Continuous Crystallization: A Study of the Polymorphs of L-Glutamic Acid
Source: Cryst Growth Des. 2023 Mar 14;23(4):2485–503. doi: 10.1021/acs.cgd.2c01424 (PMC10080659; doi:10.1021/acs.cgd.2c01424)
Supplement: Supplementary file 1 — cg2c01424_si_001.pdf [file cg2c01424_si_001.pdf]

SI: Process Performance and Operational Challenges in Continuous Crystallization: A Study of the Polymorphs of L-Glutamic Acid

Ramona Achermann, Andraž Košir, Brigitta Bodák, Luca Bosetti, and Marco Mazzotti*

Institute of Energy and Process Engineering, ETH Zurich, 8092 Zurich, Switzerland

E-mail: marco.mazzotti@ipe.mavt.ethz.ch

Phone: +41 44 632 24 56. Fax: +41 44 632 11 41

1 Population Balance Equation (PBE) Model

1.1 Secondary Nucleation Rate Expression

In the existing literature concerning the continuous crystallization of L-glutamic acid (LGA), mostly the kinetic expressions proposed by Hermanto *et al.* [1] are used (eq. 1), describing the formation of new nuclei of LGA in a secondary nucleation mechanism. In these equations, cross-nucleation is included only for the β-polymorph, meaning that nuclei of βLGA are formed in the presence of both polymorphs, i.e., αLGA and βLGA.

$$B_\alpha = k_{\mathrm{b},\alpha,0}(S_\alpha - 1)\mu_{3,\alpha} \tag{1}$$

In this work, however, also a cross-nucleation term for αLGA is introduced in the secondary nucleation rate expression, as shown in eq. 2.

$$B_\alpha = k_{b,\alpha\beta}(S_\alpha - 1)\mu_{3,\beta} + k_{b,\alpha,0}(S_\alpha - 1)\mu_{3,\alpha} \tag{2}$$

The cross-nucleation term has a rate constant $k_{b,\alpha\beta}$, which was determined to be in the order of $\approx 10^2$. This value was obtained by fitting the simulated dynamic evolution of the system conducted in the α steady-state region. In the literature, the system without considering the cross-nucleation of αLGA, but with mixed initial seeds, evolves into an α steady-state.[2] By introducing cross-nucleation of α and by seeding with pure β the simulation exhibits the same evolution towards the same steady-state with the proposed value of $k_{b,\alpha\beta} \approx 10^2$. As one can see from Figure 1, the transformation from the β product seeded initially to a production of α at steady-state always occurs, however how fast this transformation occurs depends on the actual value of $k_{b,\alpha\beta}$.[2]

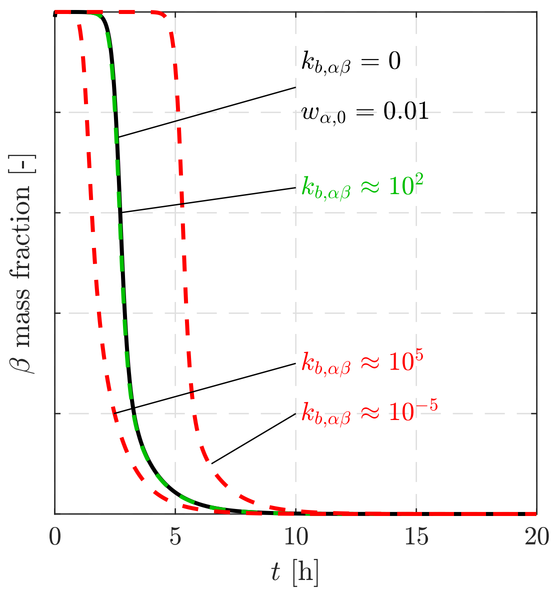

Figure 1: Simulation showing how βLGA initial seeds are transformed into αLGA via a solvent-mediated polymorphic transformation (SMPT) at α-favouring condition. The original model is shown in green[1] model and the modified nucleation model in black.

This modification is now conceptually more consistent, since the potential nucleation of αLGA in the system is accounted for in the model equations by the newly introduced cross-nucleation term. However, since the new αLGA cross-nucleation expression lacks experimental validation, the value of $k_{b,\alpha\beta}$ can indeed vary. Moreover, Figure 1 shows that already a small mass fraction

of αLGA in a suspension of βLGA crystals in an MSMPR operated in α-favouring condition leads to a shift towards the α steady-state. The presence of small amounts of αLGA particles can be the consequence of a primary nucleation event, which is stochastic and can occur during a continuously operated process, or a surface-based secondary nucleation event.[3–6] Furthermore, it can also be a consequence of having a reactor, which is not properly cleaned, or impure seeds. In fact, the latter is used in literature numerical studies, to account for potential primary nucleation, in order to end up in the expected steady-state.[2]

1.2 Solution-Fed Crystallizer: Results Sieving

The two different effects comprising a non-homogeneous withdrawal are considered in the model: dilution and sieving. Both effects can be described in a single expression with a size-independent scalar values ε and δ, whereas the size-dependent function $\omega(x)$ is used to describe the sieving effect in eq. 3–4[7].

$$\delta = 0.5(1-\varepsilon) \tag{3}$$

$$\omega(x) = 1 - e^{-ax^2} \tag{4}$$

Here, withdrawal effects are presented for a single population, however, they can be easily extended for two polymorphic populations, namely, $i \in \{\alpha, \beta\}$. The effect of both dilution and sieving can be explained by introducing an effective residence time:

$$\tau_{\text{eff}}(x) = \frac{\tau}{1-\varepsilon-\delta\omega(x)} \tag{5}$$

eq. 5 shows that larger particles, which are generally undersampled, remain longer in the crystallizer, therefore their effective residence time is larger. Same effect is caused by dilution with a positive value of $\varepsilon > 0$. Conversely, in the case of $\varepsilon < 0$, the particles are oversampled and their effective residence time is shorter, giving less time for particles to grow and worsen process

performance, characterized in the terms of KPIs.

Figure 2 shows the three KPIs for single crystallizer operated under a representative withdrawal for comparison. The influence of sieving is shown in Figure 3 for the operation of a single crystallizer. The effects observed already for the dilution effect ($\varepsilon = 0.5,\ \delta = 0$) are further amplified with an increase in productivity and yield as well as a shift towards the production of βLGA (i.e., since the thermodynamic stable polymorph is favored at higher residence times). For the sieving effect, $\delta = 0.5(1-\varepsilon)$ and $a = 5 \times 10^5$ m^{-2} were chosen.[7]

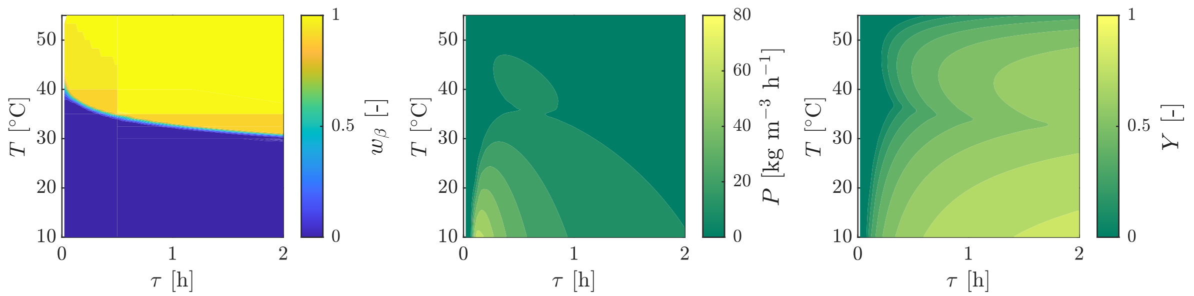

Figure 2: Contour plot showing purity w_β (*left*), productivity P (*middle*), and yield Y (*right*) as a function of temperature T and residence time τ_0 for one crystallizer operated under a representative withdrawal ($\varepsilon = 0,\ \delta = 0$).

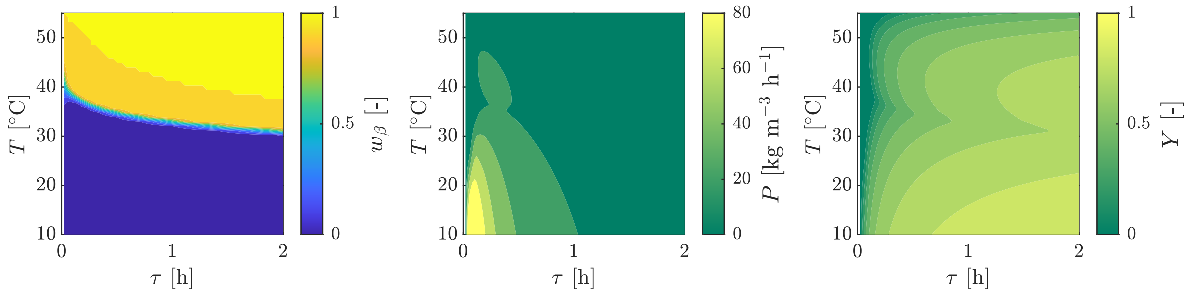

Figure 3: Contour plot showing purity w_β (*left*), productivity P (*middle*), and yield Y (*right*) as a function of temperature T and residence time τ_0 for a crystallizer operated under a non-representative withdrawal considering both a dilution and a sieving effect ($\varepsilon = 0.5,\ \delta = 0.5(1-\varepsilon)$).

1.3 Results Cascade

In Figure 4, a simulation study investigating the effect of the operating temperatures and residence times in the second MSMPR of the cascade was performed to identify potential improvements in the overall process performance. To this end, the second crystallizer is continuously fed with a population of pure βLGA crystals formed in the first crystallizer ($T_1 = 45$ °C, $c_0 = 30$ g kg^{-1}). Results are shown in Figure 4. Note that to construct the figure, $c_2^{\text{in}} = c_1^{\text{out}}$, τ_2 and $c_{\text{ss},2}$ are needed.

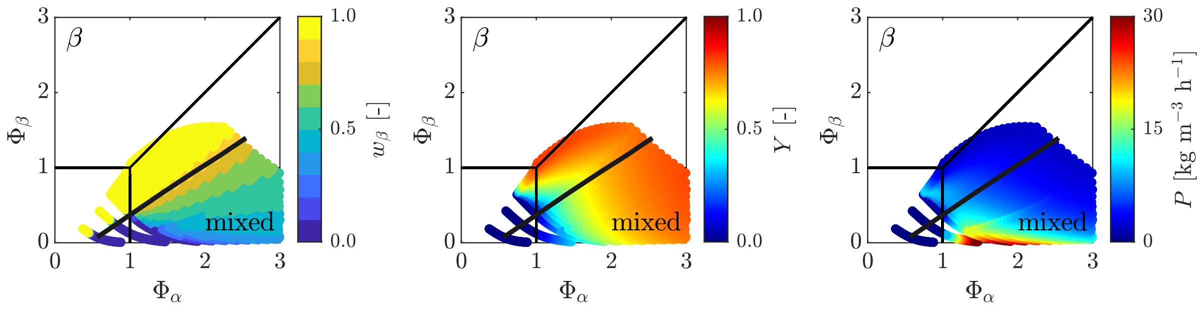

Figure 4: Heat maps showing purity of βLGA w_β (*left*), yield Y (*middle*), and productivity P (*right*) as a function of temperature T and residence time τ_0 for the second MSMPR in a cascade of two. The first MSMPR continuously seeds a population of pure βLGA crystals into the second MSMPR. Both crystallizers are operated with a representative withdrawal ($\varepsilon = 0,\ \delta = 0$). The steady-state map boundaries of a single MSMPR are shown in grey to ease comparison, whereas the black line denotes the 99% purity of βLGA.

Comparing the operation of a single MSMPR with the one of a cascade, it can be observed that βLGA can be obtained at lower temperatures (left panel in Figure 4). From the yield heat map, one can see that a clear increase is observable due to the higher residence times. Moreover, βLGA can be produced at lower temperatures thus, crystallizing out more. The increase in suspension density (i.e., leading to an increase in the third moment $\mu_{3,i,2}$) is not directly visible in the productivity. It is smaller compared to a suspension-fed crystallizer due to the need for a second vessel and corresponding higher residence times. Therefore, the suspension-fed single crystallizer clearly outperforms the cascade and the solution-fed single MSMPR in terms of productivity.

2 Experimental Study

2.1 Experimental PATs

In this section, the methodology to construct the calibration curve for XRD is shown. For the calibration, pure αLGA and βLGA crystals were produced via a pH-shift[8,9] and 13 binary mixtures including weight fractions of the α-polymorph, w_α, between 0 and 1 were prepared. Each sample was grinded before the measurement to ensure a homogeneous distribution and no bias towards a polymorph.

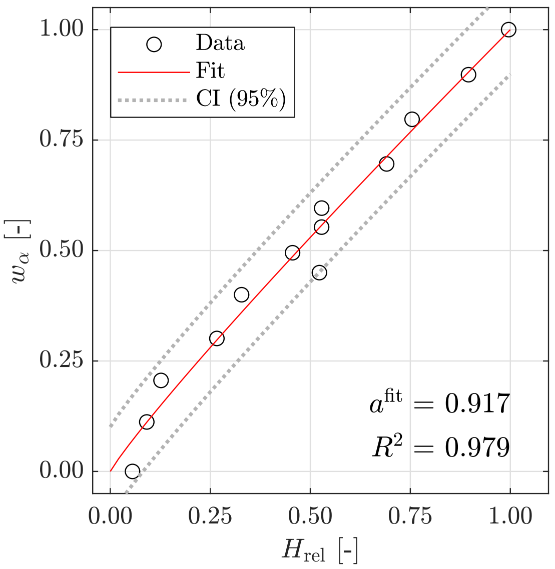

Figure 5: XRD calibration curve. A non-linear regression (shown with a red line) is conducted using an exponential correlation of the form $w_\alpha = (H_{\text{rel}})^b$, where the parameter b is fitted to the experimental data (shown with black points). The 95 % confidence interval is given by a dashed black line.

XRD Calibration: Two characteristic peaks were selected and a regression was performed. For αLGA, the characteristic peak was at $2\theta = 18.2\,^\circ$ and for βLGA at $2\theta = 10.2\,^\circ$.[2,10] An exponential function of the form $w_\alpha = H_{\text{ref}}^b$ was used for the calibration. Results are provided in Figure 5, where the following expression for fitting the data was found: $w_\alpha^{\text{XRD,exact}} = (H_{\text{rel}})^{0.917} \pm 0.10 =$

$w_{\alpha}^{\text{XRD,predict}} \pm 0.10$. Note that the standard deviation is relatively high, especially due to some measurement outliers around the 50/50 mixtures.

2.2 Concomitant Polymorphism

In both operating configurations, i.e., seeded batch reactor and MSMPR, which are two industrially relevant processes for control of polymorphism, the production of new nuclei occurs predominantly via secondary nucleation and both strategies can become ineffective due to concomitant polymorphism. Concomitant polymorphism describes different mechanisms leading to the formation of more than one polymorph at the same time. It includes SMPT, competing homogeneous nucleation of a second polymorph, and cross-nucleation.[11,12] In a batch process, in addition to the latter two phenomena[13], provided sufficient time, a SMPT eventually occurs, producing always the thermodynamically stable polymorph, even if a given meta-stable polymorph is favoured during the nucleation step. This phenomenon follows the Ostwald's rule of stages.[14] Batch processes are therefore limited in terms of time, but also in reproducibility and process control, since it is much more difficult to control the PSD in a batch process compared to a continuous.[2] In an MSMPR, the assigned residence time can prevent SMPT to proceed, however, cross-nucleation[15,16] can still occur. Cross-nucleation, is a form of heterogeneous nucleation, where a polymorph nucleates on/in the presence of a different polymorph.[17,18] This process does not follow Ostwald's rule of stage and is an activated process. The new polymorph can be of higher or lower thermodynamic stability as it is a kinetically controlled process, i.e., it depends on the relative growth rate of the two polymorphs and the rate of heterogeneous nucleation.[12,18,19]

2.3 Process Risk: Fouling

Encrustation is a process risk occurring especially during continuous crystallization as the supersaturation is constantly high. It affects not only the suspension transfer by continuously reducing the diameter of the outlet tube, thus, resulting in an increase in the outlet flow rate and clogging of the transfer lines but also the steady-state operation of the whole system. The steady-state opera-

tion was affected in two ways. First, the encrusted particles deplete supersaturation and therefore hinder the main crystallization process. Second, large encrusted solid particles sometimes detach from the surface and become part of the suspension, disturbing the system. If not controlled properly, encrustation increases the risk of process failure.[20,21] Avoiding encrustation includes applying low levels of supersaturation, avoiding primary nucleation and dead zones with low fluid velocities,[22,23] using additives, coating the surface, applying temperature cycling, or ultrasound.[20,21,23] Going to a cascade of MSMPRs in series has the advantage of a gradual temperature decrease and continuous seeding of the crystallizers in sequence, thus reducing the level of encrustation.[20] In this work, primary nucleation was strictly avoided through initial seeding and surfaces were carefully polished.

References

(1) Hermanto, M. W.; Kee, N. C.; Tan, R. B. H.; Chiu, M.-S.; Braatz, R. D. Robust Bayesian estimation of kinetics for the polymorphic transformation of L-glutamic acid crystals. *AIChE J.* **2008**, *54*, 3248–3259.

(2) Köllges, T.; Vetter, T. Polymorph Selection and Process Intensification in a Continuous Crystallization–Milling Process: A Case Study on L-Glutamic Acid Crystallized from Water. *Org. Process Res. Dev.* **2019**, *23*, 361–374.

(3) Cashell, C.; Corcoran, D.; Hodnett, B. K. Secondary nucleation of the β-polymorph of l-glutamic acid on the surface of α-form crystals. *Chem. Commun.* **2003**, *3*, 374–375.

(4) Ferrari, E. S.; Davey, R. J. Solution-mediated transformation of α to β L-glutamic acid: Rate enhancement due to secondary nucleation. *Cryst. Growth Des.* **2004**, *4*, 1061–1068.

(5) Cui, Y.; Myerson, A. S. Experimental Evaluation of Contact Secondary Nucleation Mechanisms. *Cryst. Growth Des.* **2014**, *14*, 5152–5157.

(6) Bosetti, L.; Ahn, B.; Mazzotti, M. Secondary Nucleation by Interparticle Energies. I. Thermodynamics. *Cryst. Growth Des.* **2022**, *22*, 87–97.

(7) Achermann, R.; Wiedmeyer, V.; Hosseinalipour, M. S.; Güngör, S.; Mazzotti, M. Model-based design of pressure-driven product removal from stirred suspensions. *Chem. Eng. Res. Des.* **2021**, *174*, 57–70.

(8) Ochsenbein, D. R.; Schorsch, S.; Vetter, T.; Mazzotti, M.; Morari, M. Growth rate estimation of β L-glutamic acid from online measurements of multidimensional particle size distributions and concentration. *Ind. Eng. Chem. Res.* **2014**, *53*, 9136–9148.

(9) Perini, G.; Salvatori, F.; Ochsenbein, D. R.; Mazzotti, M.; Vetter, T. Filterability prediction of needle-like crystals based on particle size and shape distribution data. *Sep. Purif. Technol.* **2019**, *211*, 768–781.

(10) Gong, W.; Wu, Y.; Lin, M.; Rohani, S. Polymorphism control of L-Glutamic acid in a single-stage and a two-stage MSMPR crystallizer by different seeding strategies. *Chem. Eng. Res. Des.* **2021**, *170*, 23–33.

(11) Desgranges, C.; Delhommelle, J. Molecular simulation of cross-nucleation between polymorphs. *J. Phys. Chem. B* **2007**, *111*, 1465–1469.

(12) Tao, J.; Jones, K. J.; Yu, L. Cross-Nucleation between L-Mannitol Polymorphs in Seeded Crystallization. *Cryst. Growth Des.* **2007**, *7*, 2410–2414.

(13) Nicoud, L.; Licordari, F.; Myerson, A. S. Polymorph control in batch seeded crystallizers. A case study with paracetamol. *CrystEngComm* **2019**, *21*, 2105–2118.

(14) Garside, J.; Davey, R. *From Molecules to Crystallizers: An Introduction to Crystallization*; Oxford Chemistry Primers; Oxford University Press, 2000.

(15) Lai, T.-T. C.; Ferguson, S.; Palmer, L.; Trout, B. L.; Myerson, A. S. Continuous Crystallization and Polymorph Dynamics in the L-Glutamic Acid System. *Org. Process Res. Dev.* **2014**, *18*, 1382–1390.

(16) Lai, T.-T. C.; Cornevin, J.; Ferguson, S.; Li, N.; Trout, B. L.; Myerson, A. S. Control of Polymorphism in Continuous Crystallization via Mixed Suspension Mixed Product Removal Systems Cascade Design. *Cryst. Growth Des.* **2015**, *15*, 3374–3382.

(17) Xu, S.; Hou, Z.; Chuai, X.; Wang, Y. Overview of Secondary Nucleation: From Fundamentals to Application. *Ind. Eng. Chem. Res.* **2020**, *59*, 18335–18356.

(18) Chen, S.; Xi, H.; Yu, L. Cross-Nucleation between ROY Polymorphs. *J. Am. Chem. Soc.* **2005**, *127*, 17439–17444.

(19) Yu, L. Nucleation of One Polymorph by Another. *J. Am. Chem. Soc.* **2003**, *125*, 6380–6381.

(20) McGlone, T.; Briggs, N. E.; Clark, C. A.; Brown, C. J.; Sefcik, J.; Florence, A. J. Oscillatory Flow Reactors (OFRs) for Continuous Manufacturing and Crystallization. *Org. Process Res. Dev.* **2015**, *19*, 1186–1202.

(21) Briggs, N. E.; Schacht, U.; Raval, V.; McGlone, T.; Sefcik, J.; Florence, A. J. Seeded Crystallization of β-L-Glutamic Acid in a Continuous Oscillatory Baffled Crystallizer. *Org. Process Res. Dev.* **2015**, *19*, 1903–1911.

(22) Zettler, H. U.; Wei, M.; Zhao, Q.; Müller-Steinhagen, H. Influence of Surface Properties and Characteristics on Fouling in Plate Heat Exchangers. *Heat Transf. Eng.* **2005**, *26*, 3–17.

(23) Acevedo, D.; Yang, X.; Liu, Y. C.; O'Connor, T. F.; Koswara, A.; Nagy, Z. K.; Madurawe, R.; Cruz, C. N. Encrustation in Continuous Pharmaceutical Crystallization Processes—A Review. *Org. Process Res. Dev.* **2019**, *23*, 1134–1142.
